# Acute myeloid leukaemia cells express high levels of androgen receptor but do not depend on androgen signaling for survival

**DOI:** 10.1038/s41375-025-02752-x

**Published:** 2025-09-11

**Authors:** Farideh Miraki-Moud, Linda Ariza-McNaughton, Thinzar KoKo, Jad Othman, Randal Stronge, Johann de Bono, Nigel Russell, Ian Thomas, Amanda Gilkes, Alan Burnett, Leandro Rodrigues Santiago, Fay Cafferty, Leo Taussig, Allan Thornhill, Simon O’Connor, Dominique Bonnet, David C. Taussig

**Affiliations:** 1 Cancer Biology, Acute Leukemia Lab, Institute of Cancer Research, Sutton, United Kingdom; 2 Hematopoietic Stem Cell Lab, The Francis Crick Institute, London, United Kingdom; 3 Department of Hematology, Royal Marsden Hospital, Sutton, United Kingdom; 4 Faculty of Medicine and Health, University of Sydney, Sydney, Australia and Department of Medical and Molecular Genetics, King’s College London, London, United Kingdom; 5 Haematology Department, Royal United Hospitals, Bath, United Kingdom; 6 Department of urology, Drug development, Institute of Cancer Research and Medical oncology, Royal Marsden Hospital, Sutton, United Kingdom; 7 Department of Hematology, Guy’s and St Thomas Hospitals NHS Trust, London, United Kingdom; 8 Centre for Trials Research, College of Biomedical & Life Sciences, Cardiff University, Cardiff, United Kingdom; 9 Department of Hematology, Division of Cancer & Genetics School of Medicine, Cardiff University, Cardiff, United Kingdom; 10 Paul O’Gorman Leukaemia Centre, University of Glasgow, Glasgow, United Kingdom; 11 Core Research Facilities - Genomics Facility, Institute of Cancer Research, Sutton, United Kingdom; 12 Clinical Trials & Stats Unit, Institute of Cancer Research, Sutton, United Kingdom; 13 Biological Service Unit, Institute of Cancer Research, Sutton, United Kingdom; 14 Department of Histopathology, Royal Marsden Hospital, Sutton, United Kingdom

**Keywords:** Translational research, Acute myeloid leukaemia

## Abstract

Male sex is associated with worse outcome in acute myeloid leukemia (AML) in many studies. We analyzed the survival of 4281 patients treated with intensive chemotherapy in the AML17 and AML19 trials based on sex. Men had a significantly lower remission rate than women. Men had a higher incidence of adverse cytogenetic features and a lower incidence of the relatively favorable *NPM1* mutation. However, male sex was an independent risk factor for survival in multi-variate analysis. We hypothesized that androgen signaling in men could worsen outcomes by protecting AML cells from chemotherapy. We demonstrated high levels of androgen receptor (AR) expression in AML across cytogenetic risk groups. We showed the AR expression was induced by IL-6 signaling in vitro and correlates with poor overall survival. Androgens had no effect on survival of primary AML cells in vitro, nor did they impact gene expression. Androgens did not protect AML cells against chemotherapy either in vitro or in vivo. Similar results were observed with estrogen signaling through estrogen receptor in vitro in AML cells. In conclusion, targeting the androgen pathway may not be a promising clinical strategy and sex hormone signaling in AML cells does not explain the poorer outcomes of men.

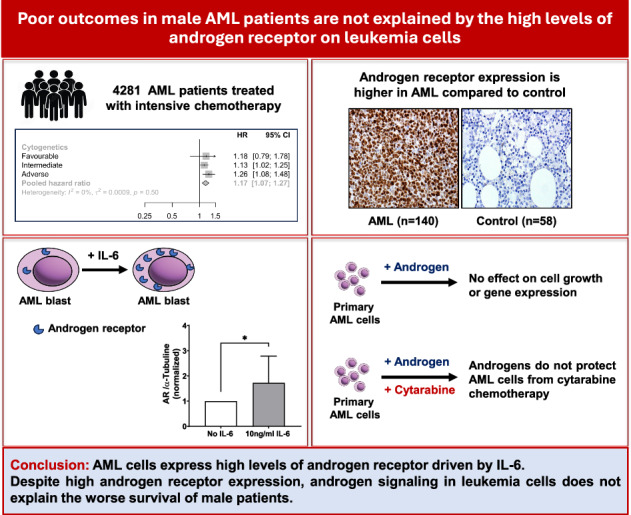

## Introduction

A number of studies suggest that men with AML treated with intensive chemotherapy have worse survival than women [1–5]. This has been highlighted in a recent perspective [6]. The incidence of mutations is different in men and women e.g. the adverse risk mutation *ASXL1* is more common in men [7, 8]. However, the poorer survival in men persists in multivariate analysis when genomic risk factors are factored [1, 3]. Male sex was an independent adverse survival factor in the AML18/HOVON-SAKK trials of intensive chemotherapy in 1910 older adults where a broad panel of prognostic genes including *ASXL1* was included. Therefore, the difference in survival between men and women is not simply an effect of more adverse genomic factors in men.

The mechanisms behind this sex difference in survival are not well understood. One potential explanation is that the differences in hormone profiles between men and women could affect the efficacy and sensitivity of leukemia cells to chemotherapy.

Testosterone and its metabolite dihydrotestosterone (DHT) bind to androgen receptor (AR) and activate AR to mediate various cellular signaling pathways. Testosterone can impact hemopoiesis. Testosterone is known to stimulate red cell production and can be used to treat anemia in hypogonadism [9] and androgens have long been used to treat aplastic anemia [10]. In addition to the pro-erythropoietic effect of testosterone there is also evidence that androgens have a role in granulopoiesis; AR is expressed in human marrow in myeloid cells [11]. AR knockout mice develop neutropenia due to reduced proliferation of precursors and decreased maturation [12]. Administering the androgen stanozolol to mice increases the number of myeloid precursors [13].

Given the role of AR signaling in granulopoiesis, we investigated whether AML might express sex hormone receptors, and gain a survival benefit from androgen signaling, potentially protecting AML cells from chemotherapy through AR signaling. Hormone blockers have a prominent role in the management of tumors that arise in sexual organs e.g. androgen blockers in prostate cancer [14] and in non-sexual tissues e.g. salivary gland carcinoma [15]. Drugs targeting these pathways are readily available and their mechanism of action has been well established. Therefore, AML dependence on AR signaling would be easy to exploit clinically.

To provide additional evidence for role of sex in survival we looked at survival data in 4281 men and women from the AML17 and AML19 NCRI clinical trials in younger adults who underwent intensive chemotherapy [16, 17]. Male sex remained an adverse factor for overall survival.

We show that AML cells express high levels of AR expression. Despite this we were unable to demonstrate any convincing effect of AR ligation or blockade on cell function. Our investigations do not suggest that hormone signaling through receptors on the AML cells is responsible for the survival advantage of women.

## Materials and methods

### Patient samples

Clinical trial data from the UK NCRI AML17/AML19 (ISRCTN55675535/78449203) studies were analyzed. Patients enrolled in AML17 and AML19 were included where there were complete cytogenetic and molecular results available.

Peripheral blood (PB) and bone marrow (BM) samples were obtained from AML patients for in vitro androgen receptor studies, and from patients with uninvolved staging marrows with a normal blood count (control group) at Royal Marsden Hospital, Sutton. The study was approved by the East of England-Cambridge South Research Ethics Committee (16/EE/0266). All studies complied with the rules of the Review Board and the revised Helsinki protocol following written informed consent. AML samples were collected at untreated presentation or relapse, and mononuclear cells were obtained by density gradient centrifugation. Plasma samples from the diagnostic BM aspirate were collected and frozen at −80 °C. Details of individual patient samples used for in vitro and in vivo studies are listed in Supplementary Tables 1 and 2.

### Statistics

Data are represented as mean ± standard deviation (SD). Significance comparing the means was calculated using appropriate *t* tests or one-way ANOVA as indicated in the figure legends. For analysis of more than two treatments two-way ANOVA was performed with Sidak’s, Dunnett’s or Tukey’s multiple comparison tests. The Kaplan–Meier method was used for estimates of overall survival, and the log-rank test to compare survival groups. Cumulative incidence of relapse (CIR) and non-relapse mortality (NRM) were calculated using cumulative incidence functions with each as the competing risk for the other, and groups compared by Gray’s test.

Additional methodological details are provided in the supplementary information.

## Results

### Clinical trial data – men have a different genomic profile to women, but male sex remains an adverse factor in multivariate analysis

**Fig. 1:**
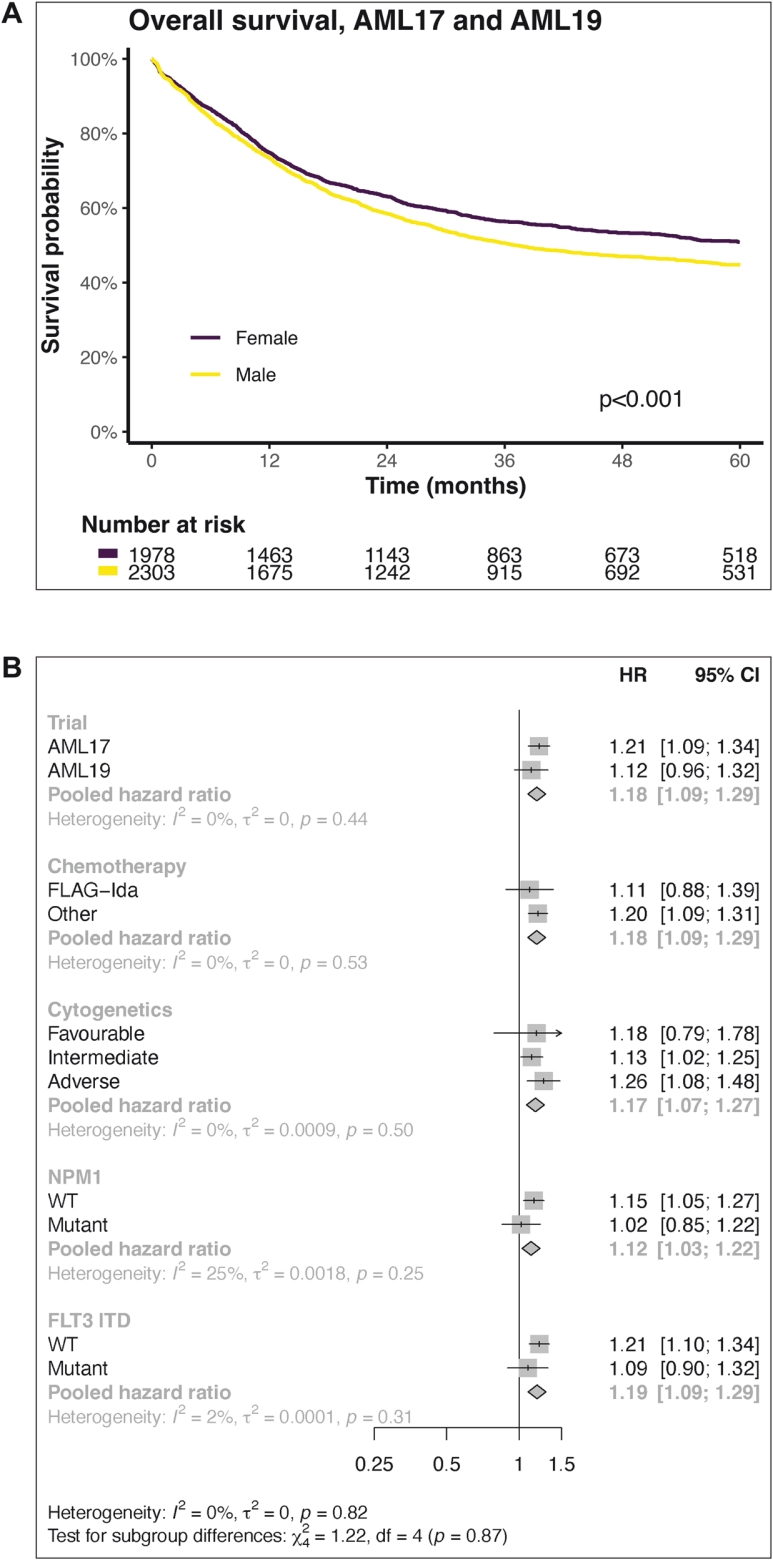
Survival by sex in NCRI AML17 and AML19 trials. **A** The overall survival by sex is displayed. **B** Forest plot of survival by subgroups with female as reference group. Men had significantly worse survival in intermediate and adverse MRC cytogenetic risk groups. ‘FLAG-Ida’ Fludarabine, Cytarabine, Granulocyte colony-stimulating factor and Idarubicin chemotherapy; ‘WT’ wild type; ‘HR’ hazard ratio; ‘CI’ confidence interval.

To assess the significance of survival between sexes in AML patients, we have used the data from the NCRI AML17 and AML19 clinical trials (*n *= 4281) in younger adults who underwent intensive chemotherapy [16, 17]. Combined data obtained from both the AML clinical trials demonstrated that the overall survival for men was poor (Fig. 1A). Men (*n *= 2303) had a significantly lower complete remission (CR)/CR with incomplete recovery (CRi) rate at 86% compared to 89% for women (*n *= 1978) (*P* = 0.006). The non-relapse mortality at 3 years was not significantly different between men (13%) and women (11%, *P* = 0.2). The cumulative incidence of relapse at 3 years was statistically higher in men though the differences are small (40% for women and 43% for men, *P* = 0.047). Therefore, inferior disease-control (higher induction failure and higher relapse) appears to be the reason for worse survival in men rather than differences in non-relapse mortality.

The incidence of cytogenetically defined risk groups was observed to be significantly different by sex. A higher percentage of males (21%) had adverse cytogenetics than females (18%) (*P* = 0.013, Supplementary Table 3). However, the incidence of intermediate risk cytogenetics was lower in men (68%) compared to women (71%). Mutational profiles also differed between sexes, with women having higher incidence of both *NPM1* and *FLT3*-ITD mutations (*P* < 0.001, Supplementary Table 3).

Men did significantly worse in all cytogenetic groups (Fig. 1B). Multi-variate analysis showed that male sex was an independent risk for worse survival (Table 1). The hazard ratio (HR) for male sex was 1.13 (95% confidence interval 1.04–1.24, *P* = 0.004). Therefore, the worse disease-control in men does not just reflect a higher incidence of adverse cytogenetic risk AML.

**Table 1:** Multivariable regression in AML patients (AML 17 and AML 19 clinical trials).

Characteristic	*N*	HR	95% CI	*p*-value
Sex				
− Female	1978	–	–	
− Male	2303	1.13	1.04 - 1.24	0.004
Trial				
− AML17	2726	–	–	
− AML19	1555	0.78	0.69 - 0.88	<0.001
Chemotherapy				
− Other	3517	–	–	
− FLAG-Ida	764	0.98	0.83 - 1.14	0.8
Cytogenetic risk				
− Intermediate	2972	–	–	
− Adverse	851	2.14	1.93 - 2.36	<0.001
− Favorable	458	0.31	0.25 - 0.38	<0.001
*NPM1*				
− Wild type	3042	–	–	
− Mutant	1239	0.56	0.50 - 0.63	<0.001
*FLT3* - ITD				
− Absent	3481	–	–	
− Present	800	1.39	1.24 - 1.55	<0.001

In order to explore whether androgen signaling in AML cells might explain the difference in outcomes between the sexes we performed laboratory experiments on a separate set of AML samples unrelated to the AML17/19 trials.

### Laboratory investigations into androgen signaling in AML cells

#### Androgen receptor is highly expressed in AML cells and associated with poor survival

We investigated whether AR is expressed in AML using immunohistochemistry (IHC) on BM trephines from 140 consecutive AML patients (MRC cytogenetic risk: 14 favorable, 94 intermediate, 14 adverse, 18 no record) and compared to 58 age-matched controls. Characteristics of AML patients are summarized in Supplementary Table 4.

**Fig. 2:**
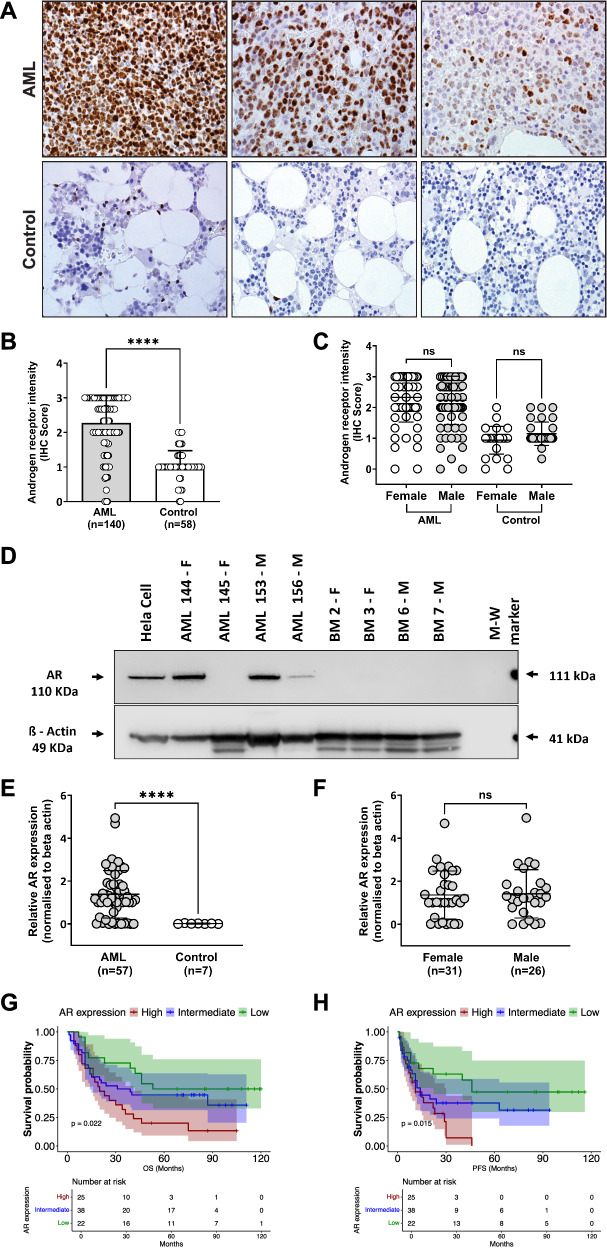
High androgen receptor expression in acute myeloid leukemia (AML) patients. **A** Representative immunohistochemistry (IHC) images of bone marrow trephine sections from three AML patients (top panels) and controls (uninvolved marrow with normal blood count; lower panels) stained for androgen receptor (AR, brown nuclear staining). Increased expression of AR is observed in the untreated AML cells. Magnification 200×. **B** Scatter plot showing AR IHC intensity score derived from AML (*n *= 140) and control (*n *= 58) and **C** between males and females. Compared to controls, AML samples showed a significant increase in AR intensity score but not between male and female groups. Each datapoint represents mean of the AR intensity score obtained from four independent observers. **D** Representative Western blot indicates that androgen receptor protein is more abundant in AMLs compared to controls. Cell lysates from primary AMLs (*n *= 57, ELN 2022 risk: 11 favorable; 22 intermediate; 24 adverse) and controls (*n *= 7) were electrophoresed using 3–8% Tris-Acetate gels and probed for AR and beta-actin. HeLa cells, positive control for AR expression. Beta actin was used as a reference protein for normalisation of protein loading. **E** Scatter plots showing the expression levels of AR assessed by Western blot. Increased levels of AR expression were observed in the AML cells with no significant difference observed between males and females (**F**). AML patients were grouped based on their AR protein levels assessed by IHC. Kaplan–Meier curves show that patients with high AR expression have worse overall survival (**G**) and progression free survival (**H**) with 95% confidence interval. Table below the Kaplan–Meier curve shows the number at risk for the indicated period of days. Statistical significance was calculated using non-parametric Mann–Whitney test (**B**, **E**, **F**) a two-way ANOVA with Tukey’s multiple comparisons between male and female groups (**C**). Error bars indicate the mean ± SD, ****P < 0.0001. ‘ns’ non-significant; ‘OS’ overall survival; ‘PFS’ progression free survival.

AR expression was significantly higher in BM from AML patients compared to controls, with no differences observed between males and females (Fig. 2A–C) or in different age groups (Supplementary Fig. 1A, B). AR expression was not significantly different in AML with *FLT3*-ITD or *NPM1* mutations or between MRC cytogenetic risk groups (Supplementary Fig. 1C–F).

AR expression was also assessed in another group of AML samples using Western blot, flow cytometry and real-time PCR. Increased levels of AR were observed in primary AML samples using all modalities with similar expression in males and females and between molecular subgroups (Fig. 2D–F, Supplementary Fig. 2A–L).

Our findings suggest that AR expression in AML patients is highly expressed and is independent of their age, sex and molecular subgroups.

We looked at the survival of AML patients treated with intensive chemotherapy (*n *= 85; MRC cytogenetic risk: 12 favorable, 63 intermediate and 10 adverse) based on their AR expression levels in BM. Patients in the high AR expression group had significantly shorter overall survival (Median OS: 23 months) and progression free survival (Median PFS: 11 months) than patients in the intermediate (Median OS: 78 months, Median PFS: 24 months) and low AR expression group (Median OS:108 months, Median PFS: 49 months) (Log-rank test, *P* = 0.022 for OS and 0.015 for PFS) (Fig. 2G, H). In multivariate analysis, high AR expressions retained independent negative prognostic significance on OS (HR: 2.66; 95% CI: 1.00–7.02; *P* = 0.04) along with MRC adverse risk group (Supplementary Table 5).

### IL-6 increases AR expression in AML cells

The cytokines interleukin-6 (IL-6) and interleukin-23 (IL-23) increase AR expression in prostate and pancreatic cancers [18–20]. To address whether IL-6 might upregulate AR in AML we measured the level of IL-6 in BM ‘plasma’ samples collected from 47 AML patients (European LeukemiaNet; ELN 2022 risk: 8 favorable; 16 intermediate; 23 adverse) and 17 age-matched controls. The level of IL-6 was significantly elevated in AML patients compared to controls (Supplementary Fig. 3A, *P* < 0.0001) with similar expression in males and females (Supplementary Fig. 3B) and between molecular subgroups (Supplementary Fig. 3C–E).

IL-23 levels in the marrow ‘plasma’ of AML patients were not significantly different to controls (Supplementary Fig. 3F–J).

**Fig. 3:**
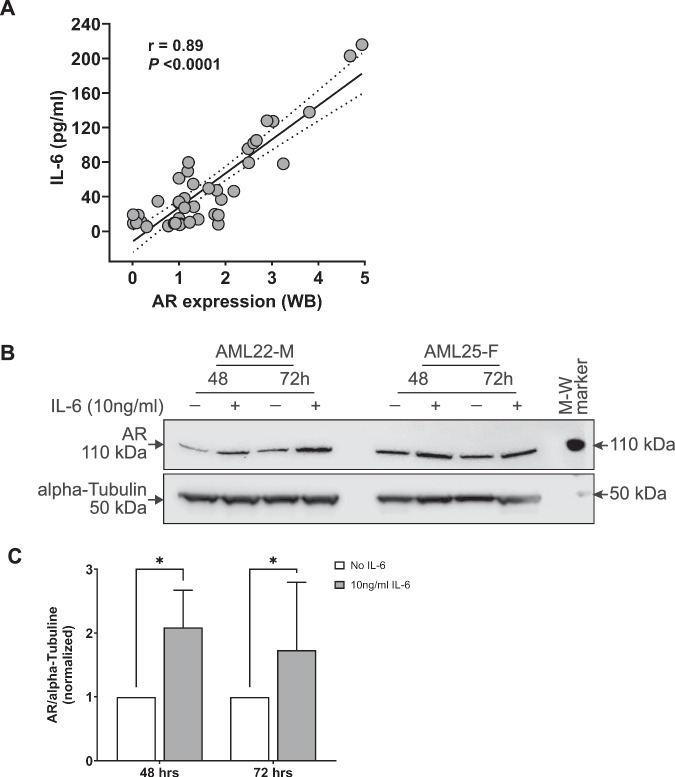
Interleukin-6 (IL-6) is associated with androgen receptor expression and increases AR levels in vitro. **A** Combined data analysis from western blot (WB) and ELISA with 42 AML patients (ELN 2022 risk: 8 favorable; 16 intermediate; 18 adverse) revealed a positive correlation between IL-6 levels and androgen receptor expression. **B** Representative western blot showing the levels of AR protein in two primary AML samples (AML22-M and AML25-F) treated with exogenous IL-6 (10 ng/ml) for the indicated period. **C** Bar plot shows the densitometric measurements obtained from primary AML samples (*n *= 13, ELN 2022 risk: 4 favorable; 3 intermediate; 6 adverse) for AR level. Statistics performed using two-way ANOVA test followed by Sidak’s multiple comparisons (**C**), Pearson correlation and linear regression test (**A**). Error bars indicate the mean ± SD; *P = 0.03. ‘M’ male; ‘F’ female; ‘M-W’ molecular weight; ‘kDa’ kilodalton.

Next, we investigated the correlation between IL-6 level and AR expression in 42 AML patients. A significant positive association of IL-6 with AR expression was observed in AML patients (*P* < 0.0001; Fig. 3A). To determine the effect of IL-6 signaling on AR expressions, primary AML samples were treated with 10 ng/ml IL-6 for 48 (*n *= 6) and 72 h (*n *= 13) and assessed by Western blotting (Fig. 3B, C). An increased expression of AR in the presence of IL-6 was observed. This suggests that the AR expression is driven by IL-6 in AML.

### Androgen has no effect on viability or growth of primary AML cells

**Fig. 4:**
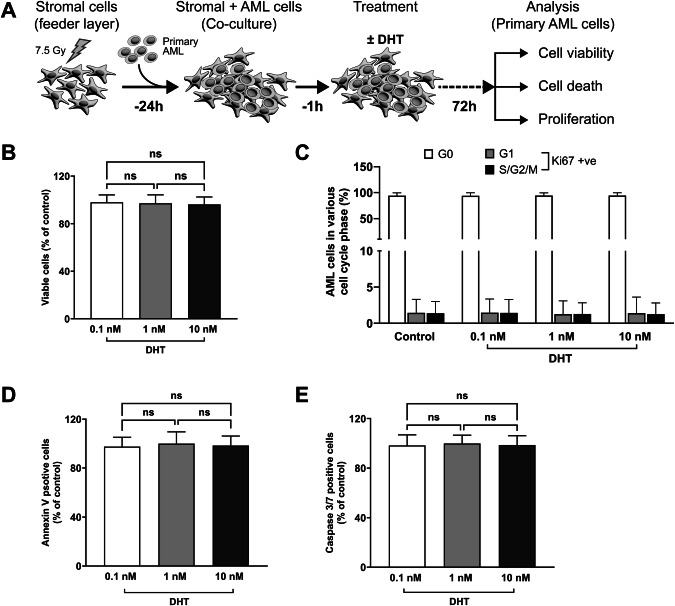
Effect of DHT on cell viability, cell death and proliferation in primary AML cells. **A** Scheme of the experimental setup. Primary AML cells were treated for 72 hours with either DMSO (control) or varying concentrations of DHT: 0.1 nM (*n *= 23), 1 nM (*n *= 23), and 10 nM (*n *= 50). Flow cytometry was used to assess several parameters: viable cell counts (**B**) using Precision Count Beads™, cell cycle distribution (**C**) using the proliferation marker Ki-67, and cell death (**D**, **E**) via Annexin V and Caspase 3/7 staining, respectively. Independent of DHT dose concentration no significant cell proliferation or change in cell cycle or cell death was observed in the treated AML cells. Statistical analysis was performed using one-way ANOVA, followed by Dunnett’s multiple comparisons test. Error bars indicate the mean ± SD in triplicates for each individual sample. ‘ns’ non-significant; ‘DHT’ dihydrotestosterone; ‘nM’ nanomolar.

To investigate the effects of androgen signaling on cell viability and growth, we cultured 50 primary AML samples (ELN 2022 risk: 11 favorable; 11 intermediate; 28 adverse) with DHT. DHT was used in preference to testosterone as the latter can be aromatized to estradiol. DHT had no significant effect on primary AML cell viability and apoptosis 72 h post treatment (Fig. 4B–E) irrespective of their sex and mutational status (Supplementary Fig. 4A–F). Longer treatment had no effect (Supplementary Fig. 4I, J). Higher doses of DHT (≥20 nM) also demonstrated no effect on growth and apoptosis of 18 primary AML cells (Supplementary Fig. 4G, H).

Finally, DHT did not significantly induce cellular senescence or alter leukemic stem cells (LSCs) frequency (Supplementary Fig. 5A–C).

### Androgen signaling has no effect on gene expression in primary AML cells in vitro

To examine the effects of androgen signaling on gene expression, we performed transcriptome analysis on 10 primary AML samples (ELN 2022 risk: 2 favorable; 6 intermediate; 2 adverse) treated either with DMSO (Control) or 10 nM DHT (Treated) for 16 h [21].

A total of 37,812 genes were analyzed demonstrating no significant differential expression between Control and Treated samples (Supplementary Fig. 6A). In terms of intra-specific expression, there were no observed differences between the samples. When assessing the overall similarity between both conditions in relation to their expression profile, the samples are grouped by patients rather than by treatment condition (Supplementary Fig. 6B). These results suggest that DHT had no significant impact on gene expression in the AML cells.

We also performed differential expression analysis with sex and *FLT3* mutation as variables. There was no significant impact of sex or *FLT3* mutation on gene expression between control and DHT treated samples (Supplementary Fig. 6C–F).

To further validate RNA-seq data, we used the same AML samples and performed real time PCR (qPCR) for five AR target genes which are known to be upregulated by androgen signaling in prostate cells (KLK2, KLK3, FKBP5, TMPRSS2 and AR) [22]. No significant differences were observed in FKBP5, TMPRSS2 and AR gene expression between control and DHT treated cells at 16, 48, and 72 h of treatment (Supplementary Fig. 7) consistent with the obtained RNA-seq data. The q-PCR results showed that AML cells do not express KLK2 and KLK3 genes.

### Androgen signaling does not protect primary AML cells from cytarabine or daunorubicin chemotherapy in vitro

**Fig. 5:**
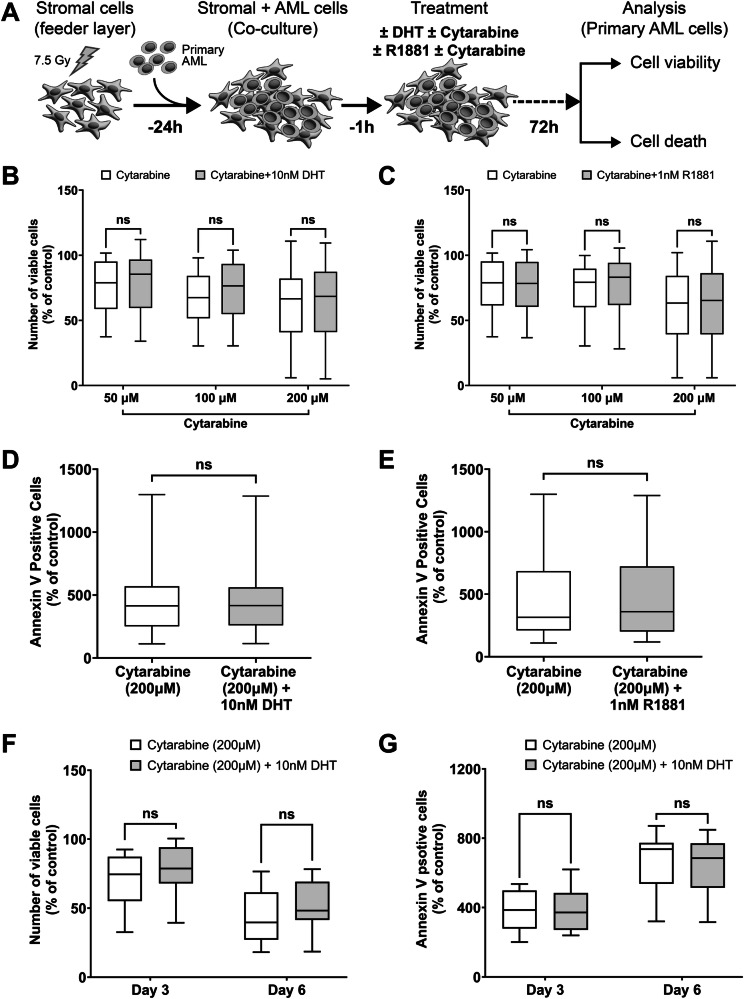
Androgens had no effect on cytotoxicity of cytarabine in primary AML cells. **A** Scheme of the experimental setup. Primary AML cells were treated either with 10 nM DHT (*n *= 62) or 1 nM R1881 (*n *= 34) in combination with indicated concentrations of cytarabine for 72 h, DMSO as a control. Number of viable cells (**B**, **C**) was determined by flow cytometry with Precision Count Beads™ and plotted as percentage of control and cell death with Annexin V staining (**D**, **E**). Prolonged treatment of primary AML cells (*n *= 12; ELN 2022 risk: 5 favorable; 5 intermediate; 2 adverse) with cytarabine (200 µM) in combination with DHT (10 nM) had no significant effect on viable cells (**F**) and apoptosis (**G**) indicating no increase in cytotoxicity. Box and whisker plots display the full range of data (minimum to maximum), as indicated by the whiskers. Statistical significance was determined using two-way ANOVA test followed by the Tukey’s multiple comparisons test. ‘ns’ non-significant; ‘nM’ nanomolar; ‘µM’ micromolar; ‘DHT’ dihydrotestosterone.

To investigate whether androgen signaling protects AML cells from chemotherapy, primary cells from 62 AML patients (ELN 2022 risk: 8 favorable; 20 intermediate; 34 adverse) were co-cultured with stromal cells and treated with three doses of cytarabine ±10 nM DHT for 72 h. There was no significant difference in the viability of AML cells treated with DHT plus chemotherapy (cytarabine or daunorubicin) compared with chemotherapy alone (Fig. 5B and Supplementary Fig. 8). We treated 34 primary AML samples (ELN 2022 risk: 4 favorable; 11 intermediate; 19 adverse) with three doses of cytarabine ±1 nM synthetic androgen (R1881). No significant effect of R1881 was seen on chemotherapy (Fig. 5C).

Longer incubation with DHT for 6 day period had no protective effect against the cytotoxicity of high-dose cytarabine (200 µM) in all the 12 primary AML samples assessed (Fig. 5F, G). Therefore, the AR ligands DHT and R1881 had no protective effect against chemotherapy induced cytotoxicity (Fig. 5D, E).

### Combination of AR blockers with cytarabine inhibits AML cell proliferation synergistically in vitro

Our data have shown higher AR expression levels in AML cells; therefore, we hypothesized that androgen signaling might protect AML cells against chemotherapy hence AR blockers (enzalutamide or darolutamide) might reduce AML cell survival. Primary AML cells were co-cultured with irradiated stromal cells and treated with DMSO as a control, 10 µM enzalutamide (*n *= 49; ELN 2022 risk: 9 favorable; 15 intermediate; 25 adverse) or 10 µM darolutamide (*n *= 65; ELN 2022 risk: 10 favorable; 18 intermediate; 37 adverse) alone, 200 µM cytarabine alone, and a combination of cytarabine with AR blockers in the presence of 10 nM DHT or 1 nM R1881. After 72 h, the number of viable and apoptotic cells was assessed. No significant apoptosis was observed following treatment with either enzalutamide or darolutamide alone (Supplementary Fig. 9A–D). Higher concentrations of darolutamide (≥30 µM) lead to increased cell death (Supplementary Fig. 9E, F).

**Fig. 6:**
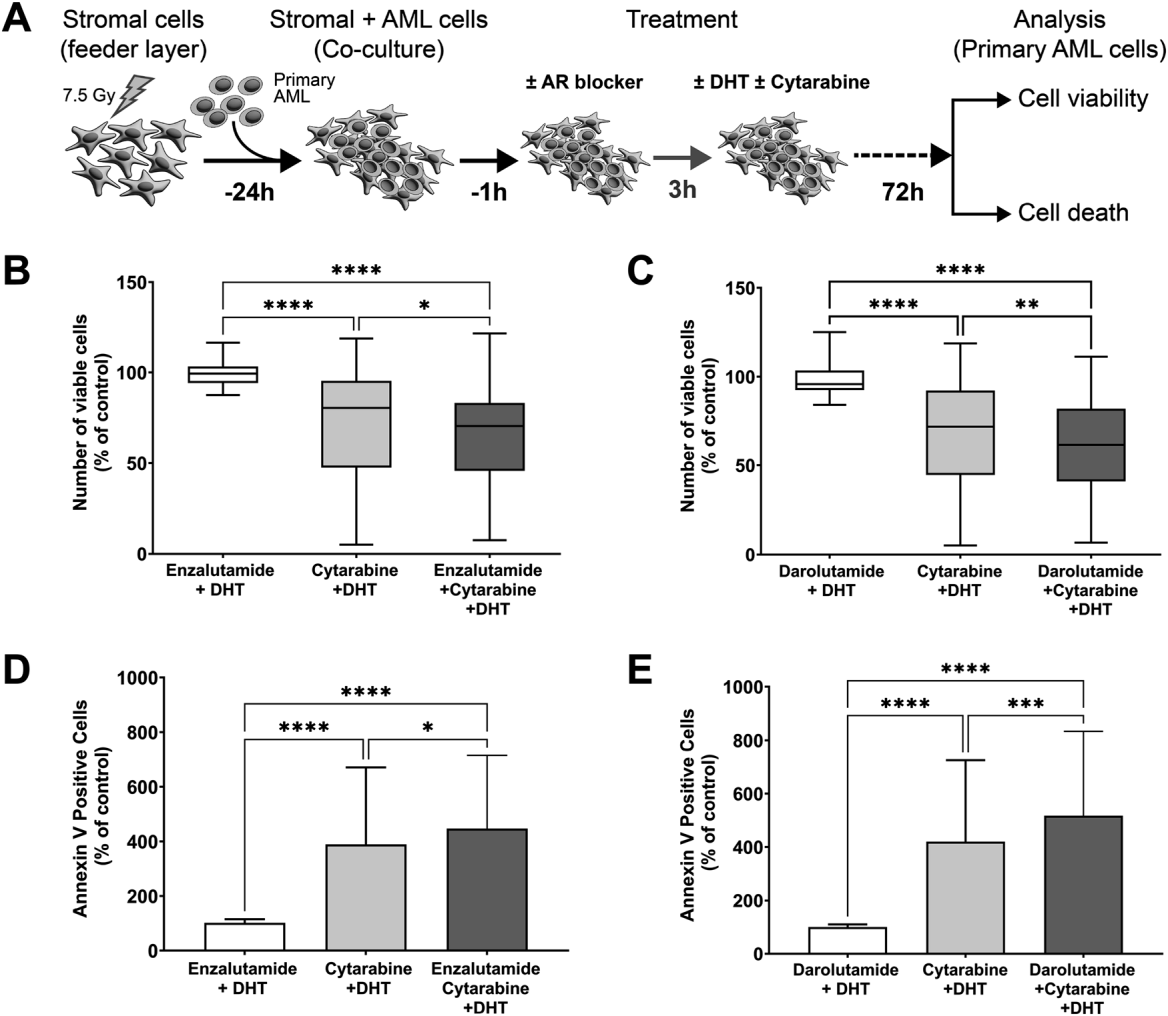
Combined treatment with AR blockers and cytarabine has an effect on primary AML cell survival. **A** Scheme of the experimental setup. Primary AML cells were treated with 10 µM androgen receptor blockers (Enzalutamide *n *= 49 or Darolutamide *n *= 65), 200 µM cytarabine and in combination in the presence of DHT (10 nM) for the indicated period of time, DMSO as a control. AR blockers when combined with cytarabine treatment significantly inhibited cell proliferation assessed by flow cytometry using Precision Count Beads™ (**B**, **C**) and increased apoptosis demonstrated by Annexin V staining (**D**, **E**). Box and Whiskers plot with the bars indicating minimum to maximum representing the sample size as mentioned above. Statistical significance was determined using two-way ANOVA test followed by the Tukey’s multiple comparisons test. *P = 0.03, **P = 0.003, ***P = 0.0002, ****P < 0.0001. ‘h’ hour; ‘DHT’ dihydrotestosterone.

The combination of AR blockers and chemotherapy resulted in a significant reduction in the number of viable AML cells and an increase in apoptosis compared to chemotherapy alone. 49 AML samples were tested with enzalutamide (Fig. 6B, D) and 65 AML samples were treated with darolutamide (Fig. 6C, E). We tested whether there was synergy between cytarabine and AR blockers using the Chou and Talalay method [23]. AR blockers synergized with cytarabine in vitro, reducing the viability of AML cells; the combination index (CI) was 0.87 and 0.81 for enzalutamide and darolutamide, respectively (CI < 1 indicates synergism). The combination of enzalutamide or darolutamide with cytarabine resulted in a reduction of viable cells only in the intermediate ELN 2022 risk group but not in adverse risk group (Supplementary Fig. 10A–D).

Primary AML cells (*n *= 20; ELN 2022 risk: 3 favorable; 3 intermediate; 14 adverse) were also treated with 50 nM daunorubicin in combination with AR blockers in the presence of DHT. In the presence of AR blockers, no enhancement in cell killing was observed following daunorubicin treatment (Supplementary Fig. 11A, B).

### Combination of AR blockers with cytarabine shows no synergism in vivo

**Fig. 7:**
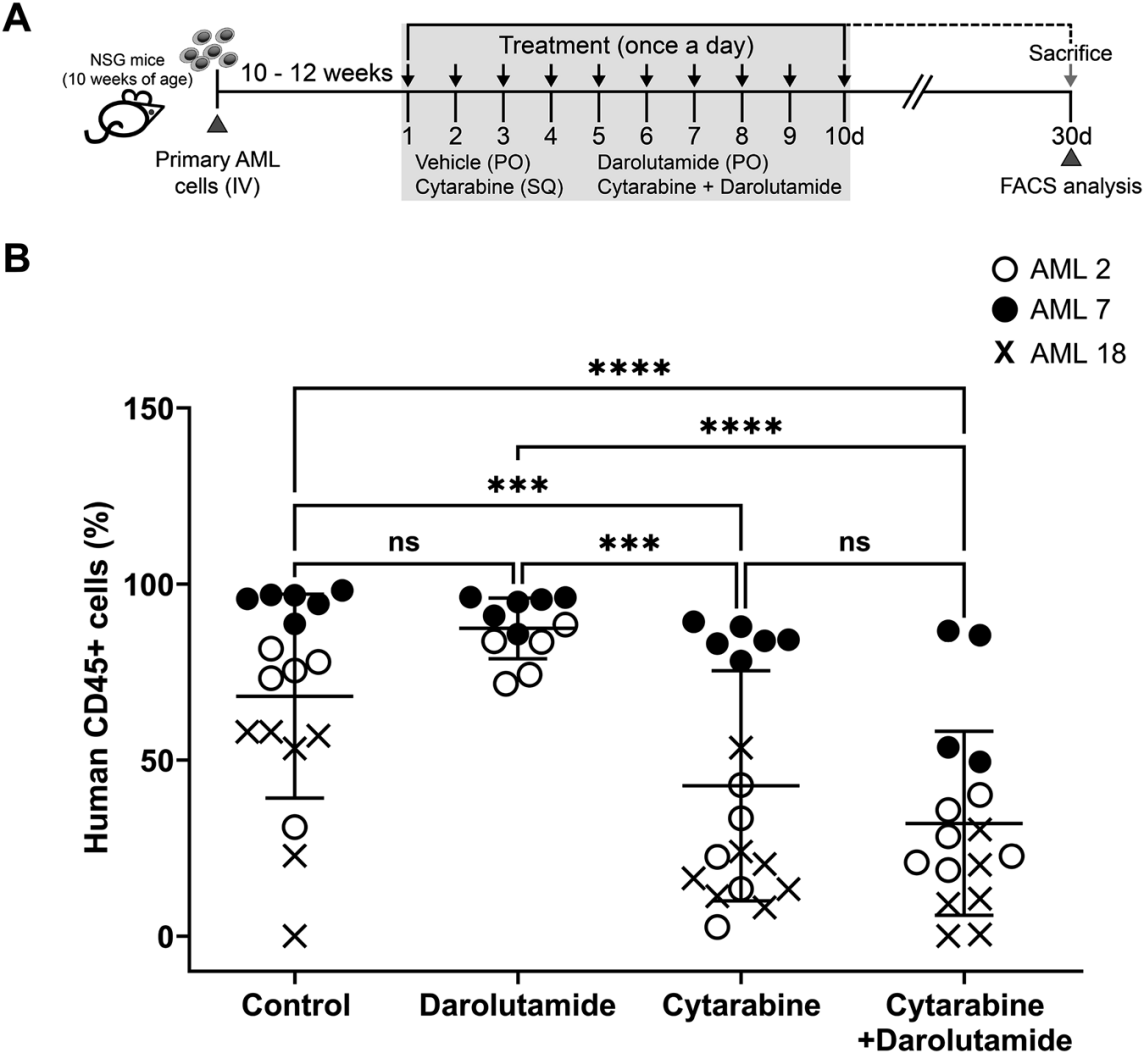
Effect of darolutamide and cytarabine in AML engrafted mice. **A** Schematic representation of treatment schedule. AML engrafted mice were established from primary AML cells (5 × 10^6^) obtained from three AML patients (AML2, AML7, AML18). AML engrafted mice for each treatment arm, for individual primary AML cells: vehicle control, *n *= 6 (administered by oral gavage, PO); darolutamide, *n *= 6 (PO; 50 mg/kg); cytarabine, *n *= 6 (administered by subcutaneous injection, SQ; 0.2 mg) and combined treatment (darolutamide + cytarabine), *n *= 6. Treatment was carried out once daily over a period of 10 days. Bone marrow from AML engrafted mice were harvested following necropsy post 4 weeks beginning initial treatment. Enumeration for human leukemia cells from these harvested AML cells were assessed by immunophenotyping. Scatter plot (**B**) shows percentage of AML cell population (human CD45+) obtained from each AML engrafted mice for the indicated treatments. Combined therapy of darolutamide and cytarabine showed no significant decrease in AML population compared to standard chemotherapy agent cytarabine. Each symbol represents individual AML patients (open circles AML2, close circles AML7, Xs AML18) and each data point represents the AML engrafted mice for indicated treatment. Statistical significance was determined using two-way ANOVA test followed by the Tukey’s multiple comparisons test. Error bars indicate the mean ± SD; ***P = 0.001; ***P < 0.0001. ‘NSG’ NOD scid gamma; ‘ns’ non-significant; ‘IV’ intravenous; ‘PO’ oral gavage; ‘SQ’ subcutaneous injection; ‘FACS’ fluorescence-activated cell sorting.

We tested whether the combination therapy of darolutamide and cytarabine has any synergistic effect on primary AML cells in a xenograft model. Three primary AML samples (ELN 2022 risk: 1 favorable; 2 intermediate - AML2, AML7, and AML18; Supplementary Table 1) were transplanted into male immunodeficient NSG mice (*n *= 62). The AML samples were chosen based on their sensitivity to cytarabine and darolutamide in vitro. The mice used were all male to provide physiological testosterone exposure. Once the AML grafts were established, 10–12 weeks post transplantation, we treated mice with vehicle, cytarabine, darolutamide and combination of cytarabine with darolutamide daily for 10 days (Fig. 7A).

Mice sacrificed four weeks post treatment, and their BM were harvested for enumeration of human leukemia cells by immunophenotyping. As expected, a lower percentage of AML populations was seen in mice treated with cytarabine alone compared to vehicle and darolutamide. No further reduction in the AML population was observed in the combination therapy of darolutamide and cytarabine compared to cytarabine alone (Fig. 7B), contrasting with the findings in vitro.

### The synergy of AR blockers with chemotherapy is not mediated through androgen signaling

We explored the discordant results between the in vitro and in vivo tests by performing the in vitro experiment using androgen blockers and chemotherapy but added an additional condition without DHT. Darolutamide and chemotherapy still had a synergistic effect when treated without DHT suggesting that this effect is an off-target and not mediated through the androgen receptor (Supplementary Fig. 12).

### AML cells express estrogen receptor beta, but not estrogen receptor alpha

Given that androgen signaling does not appear to explain the worse outcomes of men with AML, we explored estrogen receptor (ER) and progesterone receptor (PR) expression on AML cells to test whether estrogen might enhance the effect of chemotherapy.

We investigated the expression of estrogen receptor alpha (ERα), estrogen receptor beta (ERβ) and PR in AML cells. ERα was assessed in BM trephines from 70 AML patients (MRC cytogenetic risk: 8 favorable, 45 intermediate, 9 adverse, 8 no record) and 30 controls by IHC. ERβ and PR were examined in 23 AML samples (ELN 2022 risk: 3 favorable; 8 intermediate; 12 adverse) and 4 controls by Western blot. Our results indicated that ERα and PR are not expressed in BM from AML patients or controls (Supplementary Fig. 13A–C). ERβ expression levels were higher in AML samples compared to controls (Supplementary Fig. 13C, D). One potential explanation for better survival of women is that estrogen signaling might have a potential role in AML cell killing following chemotherapy.

### Estrogen has no effect on cell viability and growth in primary AML cells

To examine the effects of estrogen on cell growth, 31 primary AML samples (ELN 2022 risk: 2 favorable; 9 intermediate; 20 adverse) were treated with two doses of 17β-estradiol (E2; 10 and 100 nM) for 72 h. E2 had no impact on proliferation and apoptosis of primary AML cells (Supplementary Fig. 14A, B). To investigate if estrogen signaling enhances the cytotoxicity activity of cytarabine, 31 primary AML cells co-cultured with stromal cells and treated with DMSO as control, cytarabine (200 µM) alone and combination of cytarabine with either 10 or 100 nM of E2 for 72 h. E2 did not enhance the cytotoxic effect of cytarabine (Supplementary Fig. 14C, D) confirming that endogenous estrogen does not enhance the cytotoxic activity of cytarabine in females with AML.

## Discussion

We explored whether hormone signaling through hormone receptors on AML cells might explain the worse outcomes of men. We have demonstrated high levels of AR expression in AML cells but were unable to identify any convincing protective effects from androgen signaling either in vitro or in vivo. We were unable to show any pro-apoptotic effects of estrogen either. Therefore, AML does not follow the same mechanism as seen with some tumors (e.g. prostate, salivary) that gain benefit from androgen signaling through AR.

Our study is limited by the numbers of samples (*n *= 3) that were tested in vivo. It is possible that specific subtypes of AML may show vulnerability to androgen deprivation in vivo.

The GOELAMS group have tested androgen therapy following intensive chemotherapy for AML [24] and showed a survival advantage for patients randomized to androgen as maintenance. The lack of effect of androgens on AML cells shown here suggest that the benefit of the androgen in the GOELAMS study may have been mediated via an action on non-leukemic cells.

The worse survival of AML patients expressing high levels of AR likely reflects the association with IL-6. IL-6 levels are known to associate with worse outcomes in AML [25].

We observed a higher rate of *NPM1* and *FLT3*-ITD mutation in women as well as a higher incidence of adverse cytogenetic abnormalities in men, in line with other reports [7, 8, 26]. Although survival in *NPM1* mutant AML was not worse in men this may reflect a higher incidence of co-mutations in women such as *FLT3*-ITD and *DNMT3a* and *WT1* which are associated with worse outcomes in *NPM1* mutant AML [7, 8, 27].

HSC cycling is increased by estrogen [28]; estrogens might affect the types of mutations that arise by impacting the proliferation dynamics of normal haemopoietic cells.

Our data show a lower remission rate for men while the non-relapse mortality was similar. Men did worse within cytogenetic risk groups showing the adverse effect of male sex is not simply related to a higher incidence of adverse cytogenetic risk AMLs in men. The findings support the view that men do worse due to poorer disease control even within specific subgroups and is consistent with published data [1–5]. One of the limitations of our study is that we did not have a broad mutational profile of the patients.

While we observe worse outcomes for men in AML17/19, the *FLT3* subgroup results contrast with the RATFY study of midostaurin in *FLT3* mutant AML [29]. In AML17/19 men with *FLT3* mutation failed to demonstrate poorer outcome than women conversely in RATIFY men had worse survival. This may reflect the interaction of sex with the different therapies used alongside intensive chemotherapy in the trials, gemtuzumab ozagamicin in the NCRI trials and midostaurin in RATIFY.

Although our data support the notion that younger men treated with intensive chemotherapy have worse survival not all studies show this consistently. A study from the AMLCG/Alliance groups showed worse survival in younger men in the German cohort but not in the American cohort [7]. This may reflect treatment differences (e.g. no patients received allogeneic transplant in the AMLCG/Alliance study) and their interaction with sex.

Our trial data are not relevant to most older adults over the age of 60 years. A recent report shows that male sex is also a risk factor in intensively treated adults over 60 years [5], but male sex has not been shown to be adverse for older patients treated with low intensity regimens.

Even if the survival of men was not actually worse, the identification of high levels of AR expression on AML cells merited further exploration. The high levels of AR expression in AML may have been unconnected with sex differences in survival but nonetheless exploitable. Both men and women produce testosterone and androgen signaling may have supported AML in both sexes and been clinically targetable by androgen blockade.

One potential explanation for the worse survival of men is that men clear chemotherapy more efficiently than women even when taking into account differences in weight. However, dose intensification of anthracyclines did not further improve outcome in men compared to women in the AML17 study looking at daunorubicin 60 mg/m^2^ versus 90 mg/m^2^ (Supplementary Fig. 2; from ref. [16] AML19 DA90 vs 60) which might have been expected if higher clearance of anthracyclines in men was the cause.

Cytarabine is metabolized to inactive uracil arabinoside by cytidine deaminase. Polymorphisms in cytarabine metabolizing enzymes have been associated with differing outcomes in AML patients [30]. Females have lower cytidine deaminase activity than males [31]. Men clear cytarabine more quickly than women at 200 mg/m^2^ daily dosing [32]. A recent publication in pediatric AML shows that low cytarabine pharmacogenomic scores, which associate with low intracellular levels of the active form of cytarabine, associate with poorer outcomes [33]. Those with low pharmacogenomic scores who were treated with augmented doses of cytarabine performed better than those treated with standard dose of cytarabine. Further studies to look at differences in cytarabine clearance between men and women are warranted.

Other explanations for the sex difference need consideration such as the role of the immune system in AML [34]. Females have quantitative differences in some immune pathways [35] and these may provide a better anti-leukemic immune response.

Future trial design should take into account the potential impact of sex and its interactions with therapy [6, 26].

In summary, we did not provide any clear evidence that androgen signaling supports the survival of AML cells and therefore androgen blockade does not appear to be a promising therapeutic strategy in AML. The different genomic profile of AML in men does not fully explain the poor outcomes in men and male sex remains an independent adverse prognostic factor in younger adults treated with intensive chemotherapy.

## Supplementary information

Supplementary Material and Figures

## Data Availability

Data supporting the findings of this work are available within this paper and its Supplementary Information files. Any part of the dataset can be made available to other researchers via application to the corresponding author.
