# Supplementary material for: Acute myeloid leukaemia cells express high levels of androgen receptor but do not depend on androgen signaling for survival
Source: Leukemia. 2025 Sep 11;39(12):2895–906. doi: 10.1038/s41375-025-02752-x (PMC12634449; doi:10.1038/s41375-025-02752-x)
Supplement: Supplementary file 1 — Supplementary Material and Figures [file 41375_2025_2752_MOESM1_ESM.docx]

**Supplementary information**

**Title:**

Acute myeloid leukaemia cells express high levels of androgen receptor but do not depend on androgen signaling for survival.

**Authors:**

Farideh Miraki-Moud^1^, Linda Ariza-McNaughton^2^, Thinzar KoKo^1,3^, Jad Othman^4^, Randal Stronge^1,5^, Johann de Bono^6^, Nigel Russell^7^, Ian Thomas^8^, Amanda Gilkes^9^, Alan Burnett^10^, Leandro Rodrigues Santiago^11^, Fay Cafferty^12^, Leo Taussig^3^, Allan Thornhill^13^, Simon O’Connor^14^, Dominique Bonnet^2^ and David C. Taussig^1,3^

^1^Cancer Biology, Acute Leukemia Lab, Institute of Cancer Research, Sutton, United Kingdom. ^2^Hematopoietic Stem Cell Lab, The Francis Crick Institute, London, United Kingdom. ^3^Department of Hematology, Royal Marsden Hospital, Sutton, United Kingdom. ^4^ Faculty of Medicine and Health, University of Sydney, Sydney, Australia and Department of Medical and Molecular Genetics, King’s College London, London, United Kingdom. ^5^Haematology Department, Royal United Hospitals, Bath, United Kingdom. ^6^Department of urology, Drug development, Institute of Cancer Research and Medical oncology, Royal Marsden Hospital, Sutton, United Kingdom. ^7^Department of Hematology, Guy's and St Thomas Hospitals NHS Trust, London, United Kingdom. ^8^Centre for Trials Research, College of Biomedical & Life Sciences, Cardiff University, Cardiff, United Kingdom.  ^9^Department of Hematology, Division of Cancer & Genetics School of Medicine, Cardiff University, Cardiff, United Kingdom. ^10^Paul O’Gorman Leukaemia Centre, University of Glasgow, Glasgow, United Kingdom. ^11^Core Research Facilities - Genomics Facility, Institute of Cancer Research, Sutton, United Kingdom.  ^12^Clinical Trials & Stats Unit, Institute of Cancer Research, Sutton, United Kingdom.  ^13^Biological Service Unit, Institute of Cancer Research, Sutton, United Kingdom. ^14^Department of Histopathology, Royal Marsden Hospital, Sutton, United Kingdom.

**Corresponding author:**

David C. Taussig

Email: [david.taussig@rmh.nhs.uk](mailto:david.taussig@rmh.nhs.uk)

**Supplementary Methods**

**Clinical trial ethics information**

Clinical trial data from UK NCRI AML17 (Wales Research Ethics Committee 3 [REC 3] 08/MRE09/29, April 2009 to December 2014) and AML19 (Wales REC 3 14/WA/1056, November 2015 to November 2020) studies were analysed.

All methods were performed in accordance with the relevant guidelines and regulations.

**Immunohistochemical staining for androgen and oestrogen receptors**

Formalin-fixed, paraffin-embedded bone marrow trephines (BMT) from AML patients and control subjects (Patients with uninvolved staging marrows with a normal blood count) were sectioned at 4 µm using a microtome and placed on charged slides. Slides were oven baked for 40 minutes at 80°C before staining using the Ventana BenchMark ULTRA instrument (Roche, West Sussex, UK). The automated process involves initial deparaffinisation of the sections for 8 minutes followed by heat mediated antigen retrieval. Slides were incubated with Ventana cell conditioning 1 solution for 64 minutes at 100°C. This is followed by the application of monoclonal rabbit anti androgen receptor antibody (Clone SP107, concentration 1.30µg/ml, Roche) and monoclonal rabbit anti oestrogen receptor antibody (clone SP1, concentration 1µg/ml, Roche) for 30 minutes at 36°C. Slides are subsequently stained using Ventana OptiView DAB detection kit and Ventana OptiView Amplification kit to allow detection and visualisation of the target antibody. Nuclei were counterstained with haematoxylin for 2 minutes.

Appropriate negative and positive controls were used. Slides were scanned and analysed using Nanozoomer 2.0 HT digital slide scanner (Hamamatsu) running NDP Scan software, with an Olympus UPlan SApo 0.75 NA 40× objective lens.

AR expression and oestrogen receptor levels were quantified using semi-quantitative scoring by four independent observers and blinded to avoid bias. The percentage of positive cells was rated as follows: 0 score for no staining, 1 scores for 1-10% staining, 2 scores for 10-50% staining and 3 scores for more than 50% staining. Scores 0 and 1 were considered low, 2 as intermediate and 3 as high AR expression group.

**Primary cell culture and treatment**

Mouse stromal cell line MS-5 (DSMZ, Braunschweig, Germany) was used to support human primary AML cells. MS-5 cells were irradiated with 7.5Gy using a gamma irradiator and cultured in Alpha-Minimal Essential Media (alpha-MEM, without phenol red) supplemented with 10% heat-inactivated charcoal-stripped fetal bovine serum and penicillin-streptomycin in 24-well plates at a density of 2.5 x 10^4^ cells per milliliter in a humidified incubator containing 5% CO_2_ at 37°C. After 24 hours, AML cells co-cultured in triplicate with irradiated MS-5 at a density of 0.4 million cells per milliliter. Experimental setup involving co-cultured AML cells with androgen receptor blockers and chemotherapy agents. Initially, we treated with either enzalutamide or darolutamide (1, 5 and 10µM; androgen receptor blocker) for three hours, and then treated either with 10 nM DHT or 1 nM R1881 alone or in combination with the indicated doses of cytarabine (50, 100 and 200µM) or daunorubicin (5, 50 and 500nM). After a 72-hour post treatment incubation, the cells were harvested and stained with PE-anti human CD45 antibody (Beckman Coulter), which specifically labels AML cells. The absolute **cell counts** of live PE-anti human CD45^+^ AML **cells** was obtained by flow cytometry **using** Precision Count Beads™ **(**BioLegend UK Ltd, London, UK**).**

Cells were grown in media without phenol red (which has oestrogenic activity) and fetal calf serum was charcoal stripped to remove steroid hormones.

Analysis of IC50 for cytarabine and daunorubicin was obtained from four primary AML samples incubated in alpha-MEM with irradiated MS-5 and treated with cytarabine or daunorubicin for 72 hours.

**Analysis of apoptosis using annexin V and Vybrant FAM Caspase-3 and -7 assays**

For Annexin V assay, cells were washed using annexin V binding buffer (BD Biosciences) and incubated with annexin V-FITC (BD Biosciences) and human CD45-APC (Beckman Coulter) simultaneously for 15 minutes at room temperature in the dark. Cells were then washed and resuspended in phosphate-buffered saline (PBS) with 2% FBS and DAPI (4, 6-diamidino-2-phenylindoiole) and analysed on a Cytoflex LX cytometer (Beckman Coulter Life Science). Annexin V-positive cells (both DAPI negative and positive) were defined as apoptotic cells.

Caspase-3/7 activity was measured using the Vybrant FAM Caspase-3 and -7 assay according to the manufacturer’s instructions (Thermo Fisher Scientific, UK). Cells were analysed on a Cytoflex LX cytometer.

**Cell cycle analysis**

Cell cycle assessment was performed by Ki67 and DAPI dual staining. Cells were washed in PBS containing 2% FBS and stained with CD45-PE and Fixable Viability Dye eFluor™ 780 (Thermo Fisher Scientific, UK) for 30 min at 4^o^C. Cells were then fixed and permeabilized using the Permeabilization/Fixation Kit (BioLegend UK Ltd, London, UK) and stained with Ki67-FITC (BD Biosciences, UK) for 30 min, protected from light. DAPI (1:500 in PBS containing 2% FBS) was added prior to flow cytometric analysis.

**Animal experiments**

NOD/SCID/IL2rɣ-/- (NSG) male mice (8 -12 weeks old) were originally obtained from The Jackson Laboratory. All animal experiments were performed under the U.K Home Office project license (70/8904) in accordance with The Francis Crick Institute animal ethics committee guidance. Three AML samples chosen for *in vivo* experiments were screened initially for their ability to generate a graft in immunodeficient mice. For engraftment, each mouse received an intravenous (IV) injection of 8 × 10⁶ OKT3-treated AML cells. Ten to 12 weeks after transplantation 4 to 6 mice were randomly selected for the indicated treatment as follows. Darolutamide (ODM-201; provided by Bayer, UK) was administered by oral gavage (PO) once a day at 50mg/kg per mouse for 10 days. This dose was derived from previous studies (1).  Cytarabine was administered by subcutaneous injection (SQ) at dose of 0.2 mg daily for 10 days, the approximate dose that humans receive with the low-dose 20 mg twice-daily schedule (2).  Combination of darolutamide (PO once a day at 50mg/kg) and cytarabine (SQ, 0.2 mg daily) for 10 days. For the control groups, a vehicle solution consisting of Macrogol, Propylene glycol and 5% glucose (50:30:20, v/v/v) was administered PO once a day for 10 days. Mice were sacrificed four weeks after initiation of therapy. BM was harvested by crushing with mortar and pestle, and each mouse was processed individually. Engraftment was assessed by immunophenotyping as described before (3). For xenograft studies, analysis was carried out without blinding.

**Flow Cytometry analysis for AR**

One million cells stained with CD45-APC (Beckman Coulter) for 20 min at room temperature. After washing, cells were stained with Fixable Viability Dye eFluor™ 780 (Thermo Fisher Scientific, 1/1000 dilution) for 30 min at 4 °C. For intracellular staining, Fixation/Permeabilization buffer from FoxP3 Buffer Set (BioLegend, 1:4 dilution) was added to cells for 30 minutes then washed off with PBS. Next, cells were washed with Perm Buffer from FoxP3 Buffer Set (BioLegend, 1:10 dilution) and then stained intracellularly with androgen receptor (D6F11) rabbit mAb (PE conjugate, Cell Signaling Technology, 1:50 dilution, cat number: 8428) in the dark for overnight at 4 °C. The negative isotype control used for the anti-AR antibody was rabbit (DA1E) mAb IgG XP isotype control (PE conjugate, Cell Signaling Technology) adjusted to the same concentration as anti-AR mAb. After washing cells were resuspended in Perm Buffer and data were obtained using CytoFLEX LX (Beckman Coulter Life Science) and analysed with [FlowJo](https://www.sciencedirect.com/topics/biochemistry-genetics-and-molecular-biology/sequest) software. Median fluorescence intensity (MFI) was used for all analyses.

**Flow cytometry analysis for detection of leukemic stem cells (LSCs)**

A five-color flow cytometry panel was designed for identification of LSCs on primary AML cells co-cultured with MS-5 and treated with 10nM DHT and DMSO as control for 72 hours. Cells were incubated with CD34, CD38, CD45RA, CD33 and HLA-DR antibodies for 20 minutes at room temperature in 50µl of staining buffer (phosphate buffer saline, PBS with 2% of fetal bovine serum, FBS). Cells were then washed with staining buffer and resuspended in 4,6 diamidino-2-phenylindole (DAPI; 1:2000 from a 200 mg/ml stock) containing solution before analysis on CytoFLEX LX (Beckman Coulter Life Science). Gates were set up to exclude non-viable cells, debris and doublets. LSCs identified as CD34^+^CD38^−^CD45RA^+^CD33^+^HLA-DR^-^ population. Details on antibodies (sources, clones, fluorochromes and catalog number) are written in the Supplementary Table 7.

**Senescence-associated β-galactosidase assay**

The β-galactosidase activity was detected by flow cytometry using the CellEvent™ Senescence Green Flow Cytometry Assay Kit (Thermo Fisher Scientific) according to the manufacturer’s instructions. In brief, cells were stained with CD45-PE (Beckman Coulter) for 20 minutes at room temperature. After washing, cells were stained with Fixable Viability Dye eFluor™ 780 (Thermo Fisher Scientific, 1:1000 dilution) for 30 minutes at 4°C. Following washing, cells were resuspended in 100 µL fixation solution containing 2% paraformaldehyde for 10 minutes at room temperature. After another wash with PBS containing 2% FBS, cells were incubated with 100 µL diluted CellEvent™ Senescence Green Probe (1:1000) for 2 hours at 37°C without CO2. After washing, cells were resuspended into PBS containing 2% FBS, and data were acquired using a CytoFLEX LX (Beckman Coulter Life Sciences) and analyzed with FlowJo software. Mean fluorescence intensity (MFI) was used for all analyses.

**mRNA expression and real-time PCR**

Total RNA was extracted from primary samples with RNeasy Mini Kit (Qiagen) and cDNA was synthesized as described before (4). The expression level of genes was measured using Taqman real-time PCR assay. The 20x FAM dye labelled probe-primer mix and the 2x Universal Master Mix were purchased from Applied Biosystems. The reactions were performed in 96-well plates in triplicates using StepOne Real-Time PCR system (Applied Biosystems) in accordance with manufacturer’s guidance. Data were analysed using the 2^-∆∁t^ method after normalisation to GAPDH an endogenous control. All quantities were expressed as number of folds relative to the expression of GAPDH.

**Western blotting**

Western blot analysis was performed using the NuPAGE electrophoresis system (life Technologies, UK). Cells were lysed with CelLytic M cell Lysis Reagent (Sigma-Aldrich) supplied with protease and phosphatase inhibitor cocktails (Sigma-Aldrich) for 20 minutes on ice. After centrifuging at 12000g for 15 minutes, protein concentrations were determined by Bicinchoninic Acid (BCA) method. Fifty micrograms of extracted proteins were mixed with NuPAGE LDS Sample Buffer (Invitrogen) and boiled for 5 min. The denatured proteins were subjected to NuPAGE 3-8% Tris-Acetate gels (Invitrogen) and transferred on to polyvinylidene fluoride membrane (PVDF) using iBlot dry blotting system (Invitrogen). Membranes were blocked with 5% nonfat dried milk or 5% BSA in PBS Tween-20 (0.2%) for 1 hour at room temperature and then incubated with primary antibodies (Supplementary Table 6) overnight at 4°C. Unbound primary antibody was washed off with TBS-T (3× 10-minute washes) after which membranes were probed using appropriate horseradish peroxidase (HRP)-conjugated secondary antibodies for 1 hour at room temperature with agitation. Unbound secondary antibody was washed off with TBS-T (3× 10-minute washes). Membranes were coated in Pierce ECL Western Blotting Substrate to allow exposure of bands which were visualized using LI-COR developer (LI-COR Biotechnology - UK Ltd, Cambridge, UK). Membranes were washed and stripped with TBS-T and stripping buffer and re-probed if needed. For loading controls β-actin antibody or alpha tubulin were used.

### Enzyme-linked immunosorbent assay (ELISA)

# IL-6 and IL-23 levels measured in bone marrow plasma using ELISA MAX Deluxe Set Human kits (BioLegend, London, UK) according to the manufacturer’s protocol. Analysis was performed with the xMAP Technology by Luminex.

**RNA-Seq**

***Study subject:*** The study was conducted on 10 primary AML samples which were run in two different projects. The first project consisted of three primary AML samples (AML1, AML29 and AML30) co-cultured in triplicate with irradiated MS-5 and treated with DMSO for control and 10nM DHT. After 16 hours, AML cells were harvested, and RNA was extracted with RNeasy Mini Kit (Qiagen). In the second project eight primary AML samples (AML6, AML13, AML18, AML20, AML21, AML25, AML30 and AML31; AML30 was repeated) were split into control and treated for each patient, and the same procedure performed on the first project was applied. For each AML sample in the first project, we had three replicates with two conditions, totalizing 18 matched samples (9 controls and 9 treated). For the second project we had 16 matched samples (8 controls and 8 treated). A total of 34 samples were used for analysis. Sixteen hours treatment was used to allow sufficient time for transcription and translation of AR target genes while minimizing secondary effects or cellular adaptation that might occur with longer exposure (5, 6).

***Sequencing:*** RNA sequencing was performed by the Genomics facility at the Institute of Cancer Research (ICR). NEB RNA Ultra II library preparation-polyA kit was used to select the mRNA and from those to generate the strand specific libraries (directional kit). Illumina paired-end libraries were sequenced on a NovaSeq6000 (Illumina) using 2 x 100bp paired-end (PE) reads. Bcl2fastq software (v2.20.0, Illumina) was used for converting the raw basecall files to FASTQ format and to further demultiplex the sequencing data.

***Quality control:*** The resulting paired-end reads in FASTQ format were submitted to initial quality control with FastQC v0.11.9 (7). Trimming of the adapter content and sequence barcode were done afterwards using Trimmomatic v0.39 (8) with minimal length (MINLEN) set at 40 and HEADCROP set at 5. A new quality check was applied to check for the highest sequence content and quality. The 34 samples passed the quality control and were used for downstream analysis.

***Alignment and differential expression analysis:*** The trimmed reads were aligned to the reference genome hg38 (Ensembl release 92) using STAR v2.7.6a with default parameters (9). Expected counts at the gene level were estimated by HTSeq v0.12.4 (10). Both projects were run separately, and the table counts from each project were merged into one, which was used as input to the R package DESeq2 v1.40.2 (11) from the Bioconductor repository (12) to test for differential expression (Benjamini–Hochberg adjusted *p-value* (FDR) < 0.05) (13), accounting for sex, *FLT3* mutation and project type (1 or 2). Raw read counts were normalized by the effective library size, and dispersion factors were computed with default parameters, as described in Love *et al.* (11).

**Statistics**

Statistical analyses were performed using GraphPad Prism (v10) and R package (v4.2.2). Subgroup analyses are presented as Forest plots, with a fixed-effects model used for heterogeneity calculations. All tests were carried out two-sided. The level of significance was set to 0.05 without adjusting for multiplicity. Multivariate analyses were conducted using Cox proportional hazard regression model. Pearson correlation and linear regression test was employed to evaluate the correlation between IL-6 levels and AR expression.

### Reagents and antibodies

All chemicals and reagents were purchased either from Sigma-Aldrich (Merck Life Science UK Limited, Gillingham, UK) or Thermo Fisher Scientific. Enzalutamide (MDV3100; Cat no: S1250), darolutamide (ODM-201; Cat no: S7559), dihydrotestosterone (DHT; Cat no: S4757), cytarabine (Ara-C: Cat no: S1648), daunorubicin (Cat no: S3035) and estradiol (17beta-Estradiol: Cat no:S1709) were purchased from Selleckchem (Selleck Biotechnology Limited, UK) and R1881 (Cat no: R0908) from Sigma-Aldrich (Merck Life Science UK Limited, Dorset, UK). Darolutamide for *in-vivo* study was provided by Bayer, UK. Antibodies used for immunohistochemistry (IHC) and flow cytometry were purchased from the following companies: anti-androgen receptor (SP107) rabbit monoclonal primary antibody (Cat no: 760-4605) and anti-oestrogen receptor (SP1) rabbit monoclonal primary antibody (Cat no: 790-4325) for IHC from Roche Diagnostics Limited (West Sussex, UK). Androgen receptor (D6F11) XP rabbit mAb (PE conjugate) and rabbit (DA1E) mAb IgG XP isotype control (PE conjugate) from Cell Signaling Technology (Leiden, The Netherlands). Antibodies used in Western blot and flow cytometry are summarised in supplementary Table 6 and supplementary Table 7, respectively.

**Supplementary Figure 1. There is no apparent difference in androgen receptor expression across age groups in both control and AML subjects** **and in AML with MRC cytogenetics risk groups**. Scatter dot plot represents the mean of AR intensity score determined by four independent observers from IHC sections stained for anti-androgen receptor across various age groups among (**A**) AML patients, (**B**) controls, (**C**) AML with *FLT3*-ITD and wild-type *FLT3*, (**D**) AML with *NPM1*-mutated and wild type (*NPM1*-WT), (**E**) AML with MRC cytogenetic risk groups and (**F**) AML with cytogenetically defined subgroups. The median age for AML patients was 61 years (range 18-93 years) and 60 (range 22-91 years) for controls. AR expression was similar across all age groups for both AML patients and controls. AR expression was not statistically different between AML patients with *FLT3*-ITD and those with *FLT3*-WT, nor between AML patients with *NPM1* wild type and NPM1 mutated. There were also no significant differences in AR expression between AML patients with different MRC cytogenetic risk groups, nor in cytogenetically defined subgroups. Statistical analysis performed using ordinary one-way ANOVA test followed by the Tukey’s multiple comparisons test. Error bar represents the SD of the mean for the indicated number of patient samples (n). **P* < 0.01, *****P* < 0.0001; ‘ns’ non-significant.

**
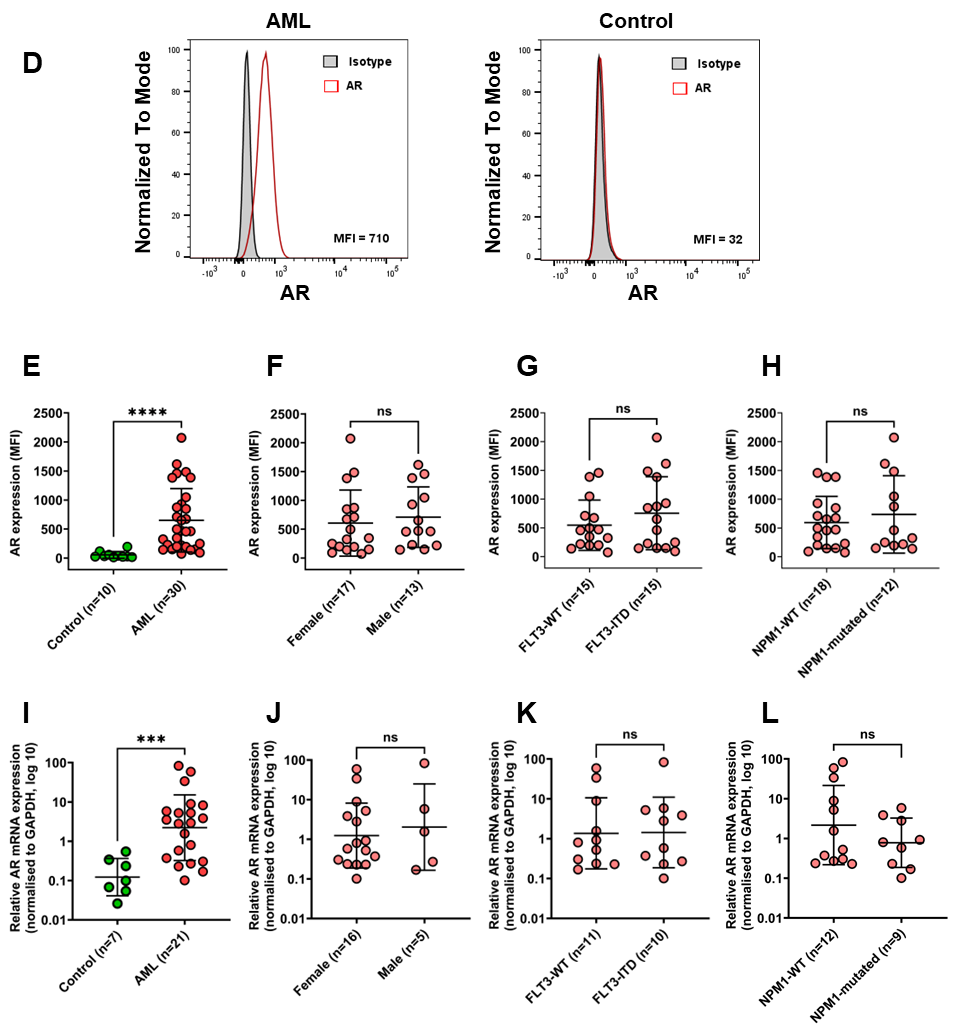
**

**Supplementary Figure 2. Androgen receptor expression levels in AML patients.**

(**A-C**) Scatter plots showing the expression levels of AR assessed by Western blot, flow cytometry (**E-H**) and qPCR (**I-L**). Increased levels of AR expression were observed in the AML patient samples, with no significant differences between males and females, *FLT3*-ITD and wild-type or between *NPM1*-mutated and wild-type. No significant differences observed between International Consensus Classification (ICC) subgroups where 3 or more samples from each molecular subgroup were tested (**C**). (**D**) Representative FACS plot showing the expression level of PE conjugated AR monoclonal antibody (Red line) compared with its isotype rabbit monoclonal IgG (Black line). The histogram shows intracellular overlay for the isotype and AR in a control (control 8) and primary AML cells (AML5). Quantitation performed relative to beta-actin (A - C), IgG isotype (D - H) and GAPDH (I - L). Statistics were performed using ordinary one-way ANOVA (A, B and C) followed by the Dunn’s multiple comparisons test and non-parametric Mann–Whitney test (E - L) comparing the levels of AR expression for the indicated number of samples (n) and. Error bars represent the mean ± SD; **P* = 0.04, ***P* = 0.002, ****P* = 0.0002, *****P* < 0.0001; ‘ns’ non-significant. ‘MFI’ mean fluorescence intensity.

**Supplementary Figure 3. Plasma IL-6 and IL-23 levels in AML patients. (A**) Scatter plots show that IL-6 level is significantly elevated in bone marrow plasma of AML patients (n = 47) compared to controls (n =17). (**B**) IL-6 levels were comparable in male and female patients with AML. (**C**) No difference observed in AMLs with *FLT3*-ITD and those with wild-type or (**D**) between *NPM1*-mutated and wild-type or (**E**) between ICC subgroups where 3 or more samples from each molecular subgroup were tested. (**F**) IL-23 level is not increased in AML patients compared to controls. (**G**) No significant differences observed between males and females, (**H**) between *FLT3*-ITD and wild-type, or (**I**) between *NPM1*-mutated and wild-type, or (**J**) between ICC subgroups where 3 or more samples from each molecular subgroup were tested. Statistics was performed using a non-parametric Mann–Whitney test (A, F) and ordinary one-way ANOVA test followed by the Dunn’s multiple comparisons test (B, C, D, E, G, H, I, J). Error bar represents the SD of the mean for the indicated number of patient samples (n). **P* = 0.02, ***P* = 0.003, ****P* = 0.0002, *****P* < 0.0001. ‘ns’ non-significant.

**Supplementary Figure 4. DHT had no effect on survival or apoptosis of primary AML cells.** (**A-C**) Primary AML samples (n = 50, ELN 2022 risk: 11 favorable; 11 intermediate; 28 adverse, 23 females and 27 males) were treated with 10nM DHT for 72hours. The number of viable cells were quantified using flow cytometry with Precision Count Beads™. (**D-F**) Apoptotic cell death measured by Annexin V and DAPI staining using flow cytometry. DHT treatment did not lead to any significant changes in cell proliferation or apoptosis between males and females, *FLT3*-ITD and wild-type, or between ICC subgroups where 3 or more samples from each molecular subgroup were tested. (**G**) Primary AML samples (n = 18, ELN 2022 risk: 3 intermediate; 15 adverse, 8 females and 10 males) were treated with higher dose of DHT (1 to 100 nM) for 72hours. Higher dose of DHT did not lead to any significant changes in cell proliferation (**G**) or apoptosis (**H**). (**I**) Primary AML cells (n = 8, ELN 2022 risk: 4 favorable; 3 intermediate; 1 adverse, 4 females and 4 males) were treated with and without 10 DHT for 3, 6 and 9 days. While overall cell growth declined after days 6 and 9, no significant differences in cell viability (**I**) or apoptosis (**J**) were observed between DHT-treated and untreated cells. Statistics performed using two-way ANOVA test followed by the Tukey’s multiple comparisons. The values are expressed as mean ± SD from triplicate samples. ‘ns’ non-significant.

**Supplementary Figure 5. DHT had no effect on leukemic stem cell (LSC) frequency or cellular senescence of primary AML cells.** (**A**) The impact of androgen signaling on LSC frequency was tested in 30 primary AML samples (ELN 2022 risk: 6 favorable; 10 intermediate; 14 adverse). AML cells were cultured with 10nM DHT or DMSO for 72 hours.  LSCs were defined phenotypically as the CD34+CD38−CD45RA+CD33+HLA-DR- population. The gating strategy used to define LSCs in AML153 is shown following treatment with 10nM DHT (lower panel) or DMSO as a control (top panel). (**B**) DHT did not result in a significant change in the frequency of LSCs compared to DMSO treated. (**C**) The impact of androgen signaling on senescence was tested in 34 primary AML samples (ELN 2022 risk: 7 favorable; 13 intermediate; 14 adverse). AML cells were treated with 10nM DHT or DMSO for 72 hours and senescence associated-β-galactosidase (SA-β-gal) activity measured by flow cytometry and expressed as the mean fluorescence intensity (MFI).  DHT did not exert a significant effect on the induction of cellular senescence in AML cells.  Statistics performed using the parametric t-test. The values are expressed as mean ± SD from duplicate samples. ‘ns’ non-significant.

**
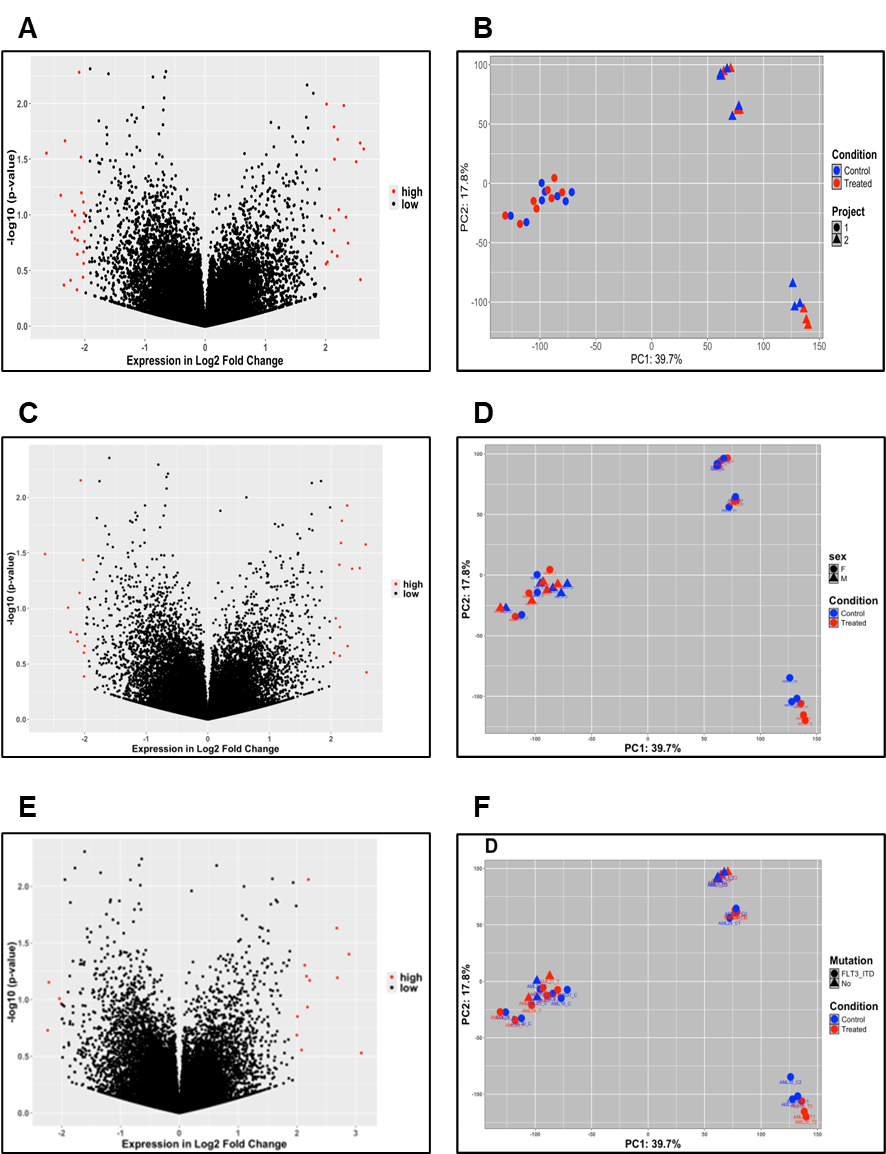
**

**Supplementary Figure *6*. RNA-seq data from primary AML cells (n = 10) treated with DHT compared with control.** (**A**) Volcano Plot from differential expression analysis between Control and DHT (10nM) treated primary AML cells (n = 10; AML1, AML6, AML13, AML18, AML20, AML21, AML25, AML29, AML30, AML31, AML30 was repeated in two different experiments). There was no gene with significant differential expression. Positive values on the x-axis represent genes more highly expressed in Control, whereas negative values show the genes more highly expressed in Treated. (**B**) PCA to visualize sample-to-sample distances as variance stabilizing transformation of the expression values (mean of normalized counts) between control and DHT treated samples. Samples were grouped by project and patient. The PCA shows that treatment with DHT is not the determinant of the clustering. Differential expression analysis accounting for sex (**C,** **D**) and *FLT3* mutation (**E,** **F**) shows no significant impact of sex or *FLT3* mutation on the differential gene expression between control and DHT treated samples. Genes with Log2 Fold Change greater than 2 or lower than -2 are highlighted in red, whereas the remaining genes are highlighted in black. ‘F’ Female, ‘M’ Male.

**Supplementary Figure 7. Validation of RNA-seq data using real time PCR (qPCR).** Five genes were chosen to validate the RNA-Seq data by qPCR in the same primary AML cells. (n = 10; AML1, AML6, AML13, AML18, AML20, AML21, AML25, AML29, AML30, AML31). KLK2 and KLK3 genes were not expressed in the primary AML cells. No significant differences observed for AR (**A**), TMPRSS2 (**B**) and FKBP5 (**C**) gene expression in control vs DHT (10nM) treated cells for indicated time points. Gene expression levels were normalized to the housekeeping gene (GAPDH). The values are expressed as mean ± SD from triplicate samples. Statistical significance was determined using two-way ANOVA test followed by the Sidak test for multiple comparisons. ‘ns’ non-significant.


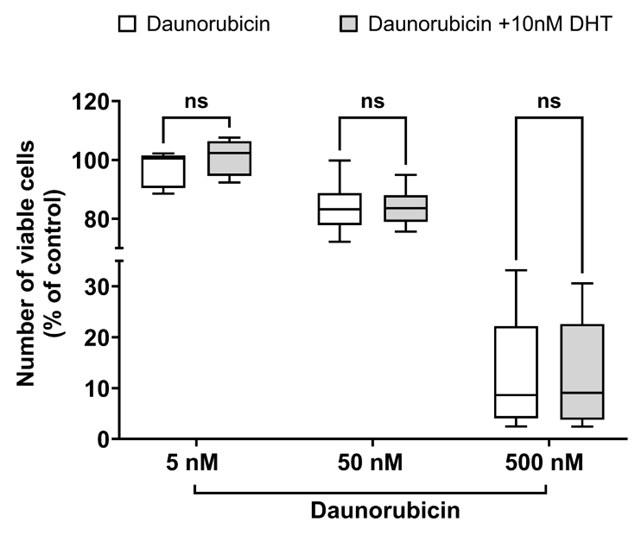


**Supplementary Figure 8. DHT had no effect on cytotoxicity of daunorubicin in primary AML cells.** (**A**) Primary AML samples (n = 10, ELN 2022 risk: 3 favorable; 5 intermediate; 2 adverse, 6 females and 4 males) were treated with 10nM DHT in combination with indicated concentrations of daunorubicin for 72h, DMSO as a control. Number of viable cells was determined by flow cytometry with Precision Count Beads™ and plotted as percentage of control. There was no significant difference in the viability of AML cells treated with DHT plus daunorubicin compared with daunorubicin alone. Box and whisker plots display the full range of data (minimum to maximum), as indicated by the whiskers. Statistical significance was determined using two-way ANOVA test followed by the Tukey’s multiple comparisons test. ‘ns’ non-significant.

**Supplementary Figure 9. AR blockers alone at 10µM had no impact on the survival and apoptosis of AML cells when used without chemotherapy.** (**A, C**) Primary AML cells were treated with 10µM androgen receptor blockers (n = 49 AMLs tested with enzalutamide or n = 65 AMLs tested with darolutamide) in the presence of 10nM DHT for 72hours. The number of viable cells obtained by flow cytometry using Precision Count Beads™ and calculated as percent from control. (**B**, **D, F**) Apoptotic cell death measured by Annexin V and DAPI staining using flow cytometry. (**E**) Primary AML cells (n = 14; ELN 2022 risk: 1 favorable; 3 intermediate; 10 adverse) treated with DMSO as control, various concentrations of darolutamide in the presence of 10nM DHT for 72hours. The number of viable cells obtained by flow cytometry using Precision Count Beads™ and calculated as percent from control. Darolutamide reduced viability of AML cells at 30 µM concentration. Statistical significance was determined using one-way ANOVA test followed by Dunnett multiple comparisons test (E, F) and paired t-test (A-D). Error bars indicate the mean ± SD; **P* = 0.03, ***P* = 0.01, ****P <* 0.0001, ‘ns’ non-significant.

**Supplementary Figure 10. Combination of AR blockers with cytarabine reduced the number of viable cells on AML patients with intermediate ELN 2022 risk group.** Primary AML cells were treated with 10µM androgen receptor blockers (enzalutamide n = 49 or darolutamide n = 65), 200µM cytarabine and in combination in the presence of DHT (10nM), DMSO as a control. AR blockers combined with cytarabine treatment significantly inhibited cell proliferation in AML patients with intermediate ELN 2022 risk groups (**A, C**) not in adverse risk (**B**, **D**). Box and Whiskers plot with the bars indicating min to max representing the sample size as mentioned above. Statistical significance was determined using one-way ANOVA test followed by the Tukey’s multiple comparisons test. **P* = 0.03, ***P* = 0.005, ****P* = 0.0006, *****P* < 0.0001; ‘ns’ non-significant.

**Supplementary Figure 11. Combined treatment with AR blockers and daunorubicin has no effect on primary AML cell survival.** Primary AML cells (n = 20; ELN 2022 risk: 3 favorable; 3 intermediate; 14 adverse) were treated with 10µM enzalutamide (**A**) or darolutamide (**B**), 50nM daunorubicin and in combination in the presence of DHT (10nM) for 72 hours. DMSO used as a control. AR blockers when combined with daunorubicin treatment had no effect on cell proliferation assessed by flow cytometry using Precision Count Beads™. Box and Whiskers plot with the bars indicating min to max representing the sample size as mentioned above. Statistical significance was determined using two-way ANOVA test followed by the Tukey’s multiple comparisons test. *****P* < 0.0001; ‘ns’ non-significant.

**Supplementary Figure 12. Darolutamide shows an off-target effect *in vitro* in the absence of androgen*.*** Primary AML cells (n = 22; ELN 2022 risk: 5 favorable; 4 intermediate; 13 adverse) were treated with androgen receptor blocker darolutamide (10µM), cytarabine (200µM) and in combination ± DHT (10nM) for 72h. DMSO as a control. AR blockers when combined with cytarabine treatment significantly inhibited cell viability assessed by flow cytometry using Precision Count Beads™. The values are expressed as mean ± SD from triplicate samples. Data were analysed by two-way ANOVA followed by the Sidak test for multiple comparisons. ***P* = 0.01 compared to control, **P* = 0.03, *** *P* = 0.0001; ‘ns’ non-significant.

**
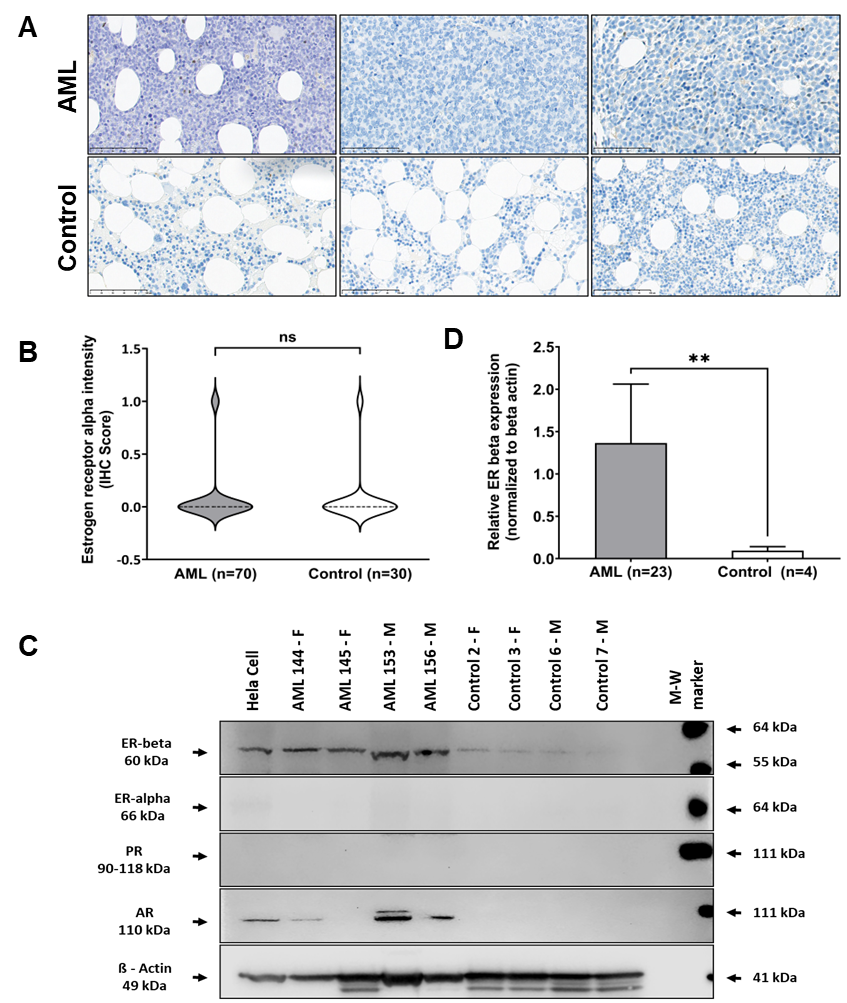
**

**Supplementary Figure 13. oestrogen receptor beta but not oestrogen receptor alpha is expressed in AML.** Representative immunohistochemical staining of oestrogen receptor alpha (ERα) and western blot analysis of oestrogen receptor beta (ERβ) expression. (**A**) IHC staining of ERα in bone marrow trephines showing absence or weak expression in AML patients (n = 70; top panels) and controls (n = 30; lower panels), magnification × 200. (**B**) Violin plot, IHC staining scores for ERα in AML and control bone marrow trephines. (**C**) Western blot analysis indicates a significant expression of AR and ERβ, whereas no apparent expression of ERα and progesterone receptor (PR) were observed in primary AML cells (n = 23; ELN 2022 risk: 3 favorable; 8 intermediate; 12 adverse). Cell lysates from primary AMLs and controls were electrophoresed using 3-8% Tris-Acetate gels and probed for ERβ, ERα, AR, PR, and beta actin. Hela cells used as a positive control for ERβ expression. Beta actin was used as a reference protein for normalisation of protein loading. (**D**) Image J was used to obtain densitometric measures from independent membranes. Data were analysed using a parametric unpaired t-test comparing ERβ expression in AML vs controls. Error bars mean ± SD, ***P =* 0*.*001; ‘ns’ non-significant. Scale bars: 100 µm.

**Supplementary Figure 14. No apparent effect was observed in primary AML cells post exogenous oestrogen treatment.** (**A**) Primary AML samples (n = 31; ELN 2022 risk: 2 favorable; 9 intermediate; 20 adverse) were treated with 17β-estradiol (E2), 10nM and 100nM for 72hours. The number of viable cells was counted by flow cytometry using Precision Count Beads™ and calculated as percentage of control. (**B**) Apoptotic cell death measured by Annexin V and DAPI staining using flow cytometry. Statistical analysis was done by paired student t-test. (**C**) Primary AML samples (n = 31; ELN 2022 risk: 2 favorable; 9 intermediate; 20 adverse) treated with 200µM cytarabine alone or combined treatment of cytarabine with E2 (10nM, 100nM). The number of viable cells post 72h were counted using flow cytometry (Precision Count Beads™) and calculated as percentage of control (DMSO). (**D**) Apoptosis analysed using Annexin V FITC and DAPI by flow cytometry. Statistical analysis was done by one-way ANOVA test. The values are expressed as mean ± SD. ‘ns’ non-significant.

**Supplementary Table 1.** Cytogenetic and molecular characteristics of acute myeloid leukemia patients used for *in vitro* and *in vivo* studies.

(*AML samples used for the *in vivo* study)

ICC: International Consensus Classification

ELN: European LeukemiaNet

| AML | Age | sex | Cytogenetics | Point Mutations and other genomic information | ICC 2022 | ELN 2022 risk |
| --- | --- | --- | --- | --- | --- | --- |
| AML1 | 41 | F | t(8;21) (q22;q22.1) | *KIT* | AML with *RUNX1::RUNX1T1* fusion | Favorable |
| AML84 | 63 | M | t(8;21) (q22;q22.1) | *FLT3*-TKD | AML with *RUNX1::RUNX1T1* fusion | Favorable |
| AML142 | 36 | M | t(8;21) (q22;q22.1) | *KIT* | AML with *RUNX1::RUNX1T1* fusion | Favorable |
| AML21 | 34 | F | inv(16) (p13;q22) | No mutations | AML with *CBFB::MYH11* fusion | Favorable |
| AML22 | 60 | M | inv(16) (p13.1;q22) | No mutations | AML with *CBFB::MYH11* fusion | Favorable |
| AML80 | 52 | F | inv(16) (p13;q22), +22 | No mutations | AML with *CBFB::MYH11* fusion | Favorable |
| AML159 | 62 | M | inv(16) (p13;q22) | No mutations | AML with *CBFB::MYH11* fusion | Favorable |
| AML160 | 50 | M | inv(16) (p13;q22) | No mutations | AML with *CBFB::MYH11* fusion | Favorable |
| AML34 | 49 | M | Normal | *CEBPA* biallelic | AML with in-frame bZIP *CEBPA* mutations | Favorable |
| AML64 | 24 | M | Normal | *CEBPA* biallelic, *FLT3*-ITD, *WT1* | AML with in-frame bZIP *CEBPA* mutations | Favorable |
| AML3 | 47 | M | Normal | *NPM1, IDH1* | AML with mutated *NPM1* | Favorable |
| AML16 | 72 | F | Normal | *NPM1, IDH1* | AML with mutated *NPM1* | Favorable |
| AML26 | 73 | F | Normal | *NPM1, IDH2* | AML with mutated *NPM1* | Favorable |
| AML54 | 51 | M | Normal | *NPM1, DNMT3A* | AML with mutated *NPM1* | Favorable |
| AML60 | 44 | F | Normal | *NPM1, WT1* | AML with mutated *NPM1* | Favorable |
| AML40 | 40 | F | Normal | *NPM1* | AML with mutated *NPM1* | Favorable |
| AML95 | 65 | M | Normal | *NPM1* | AML with mutated *NPM1* | Favorable |
| AML45 | 72 | M | Normal | *NPM1, FLT3-*ITD | AML with mutated *NPM1* | Intermediate |
| AML47 | 55 | F | Normal | *NPM1, FLT3*-TKD, *IDH1, DNMT3A* | AML with mutated *NPM1* | Intermediate |
| AML30 | 69 | F | Normal | *NPM1*, *FLT3*-ITD and TKD | AML with mutated *NPM1* | Intermediate |
| AML4 | 22 | F | Normal | *NPM1, FLT3*-ITD | AML with mutated *NPM1* | Intermediate |
| AML7^*^ | 57 | F | Normal | *NPM1, FLT3*-ITD | AML with mutated *NPM1* | Intermediate |
| AML9 | 66 | M | Normal | *NPM1, FLT3*-ITD | AML with mutated *NPM1* | Intermediate |
| AML12 | 75 | F | Normal | *NPM1, FLT3*-ITD | AML with mutated *NPM1* | Intermediate |
| AML18^*^ | 64 | M | Normal | *NPM1, FLT3*-ITD, *IDH1* | AML with mutated *NPM1* | Intermediate |
| AML67 | 64 | F | Normal | *NPM1, FLT3-*ITD*, DNMT3A* | AML with mutated *NPM1* | Intermediate |
| AML70 | 60 | M | Normal | *NPM1, FLT3-*TKD*, RAD21, WT1* | AML with mutated *NPM1* | Intermediate |
| AML74 | 75 | M | Normal | *NPM1, FLT3-*ITD*, JAK2, DNMT3A, TET2* | AML with mutated *NPM1* | Intermediate |
| AML90 | 73 | F | Normal | *NPM1, FLT3-*ITD*, DNMT3A, KRAS* | AML with mutated *NPM1* | Intermediate |
| AML153 | 48 | M | Normal | *NPM1, FLT3*-ITD*, DNMT3A, TET2,* | AML with mutated *NPM1* | Intermediate |
| AML148 | 78 | F | Normal | *NPM1, FLT3*-ITD, *DNMT3A, RAD21* | AML with mutated *NPM1* | Intermediate |
| AML156 | 55 | M | Normal | *NPM1, FLT3-*TKD, *IDH2, KIT, RAD21* | AML with mutated *NPM1* | Intermediate |
| AML31 | 59 | M | Normal | *NPM1, FLT3*-ITD, *RUNX1* | AML with mutated *NPM1* | Intermediate |
| AML2^*^ | 69 | F | Trisomy 11 | *NPM1, IDH1* | AML with mutated *NPM1* | Favorable |
| AML28 | 65 | M | Trisomy 8 | *NPM1, IDH1, CEBPA* | AML with mutated *NPM1* | Favorable |
| AML29 | 67 | F | t(1;15;9)(q21;q22;q34) | *NPM1*, *FLT3*-ITD and TKD | AML with mutated *NPM1* | Intermediate |
| AML11 | 56 | M | Normal | *FLT3*-ITD*, DNMT3A, RAD21* | AML, not otherwise specified (AML, NOS) | Intermediate |
| AML17 | 75 | M | Normal | *FLT3*-ITD | AML, not otherwise specified (AML, NOS) | Intermediate |
| AML23 | 43 | F | Normal | *FLT3*-ITD, *IDH2, DNMT3A* | AML, not otherwise specified (AML, NOS) | Intermediate |
| AML36 | 50 | M | t(2;17)(p21;q25) | *FLT3*-ITD | AML, not otherwise specified (AML, NOS) | Intermediate |
| AML72 | 52 | F | Normal | *FLT3-*ITD/TKD, *DNMT3A, KRAS,* *TET2, WT1* | AML, not otherwise specified (AML, NOS) | Intermediate |
| AML77 | 57 | F | Normal | *TET2* | AML, not otherwise specified (AML, NOS) | Intermediate |
| AML82 | 28 | F | Normal | *FLT3*-TKD, *PTPN11, WT1* | AML, not otherwise specified (AML, NOS) | Intermediate |
| AML86 | 76 | F | Monosomy X | *FLT3*-TKD | AML, not otherwise specified (AML, NOS) | Intermediate |
| AML91 | 63 | M | Normal | *DDX41* | AML, not otherwise specified (AML, NOS) | Intermediate |
| AML104 | 69 | F | Normal | *DMT3A* | AML, not otherwise specified (AML, NOS) | Intermediate |
| AML119 | 42 | F | Normal | *TET2* | AML, not otherwise specified (AML, NOS) | Intermediate |
| AML139 | 53 | F | t(1;10) (p1;p11.1), del9q | *WT1, DNMT3A* | AML, not otherwise specified (AML, NOS) | Intermediate |
| AML144 | 62 | F | Normal | *DNMT3A* | AML, not otherwise specified (AML, NOS) | Intermediate |
| AML158 | 65 | M | t(7;10) (q22;p1) | No mutations, No loss of 7 on FISH | AML, not otherwise specified (AML, NOS) | Intermediate |
| AML5 | 81 | F | Normal | *IDH2,* cryptic deletion of chromosome 7q | AML with MR cytogenetic abnormalities | Intermediate |
| AML6 | 35 | M | Trisomy 8 | *FLT3*-ITD*, DNMT3A* | AML with MR cytogenetic abnormalities | Intermediate |
| AML13 | 43 | M | t(9;11)(p21.3;q23) | *FLT3*-ITD, *KMT2A*-rearrangement | AML with *KMT2A::MLLT3* | Intermediate |
| AML44 | 39 | F | t(9;11)(p21.3;q23.3) | *KMT2A*-rearrangement | AML with *KMT2A::MLLT3* | Intermediate |
| AML19 | 78 | M | Normal | *FLT3*-ITD, *RUNX1* | AML with MR gene mutations | Adverse |
| AML20 | 72 | M | Normal | *FLT3*-ITD, *RUNX1* | AML with MR gene mutations | Adverse |
| AML8 | 71 | F | Normal | *IDH2, SRSF2* | AML with MR gene mutations | Adverse |
| AML10 | 81 | F | Normal | *SRSF2, TET2, MYC* | AML with MR gene mutations | Adverse |
| AML15 | 70 | M | Trisomy 13 | *FLT3*-ITD, *RUNX1, STAG2* | AML with MR gene mutations | Adverse |
| AML25 | 82 | F | i(22q) | *ASXL1, KMT2A-PTD* | AML with MR gene mutations | Adverse |
| AML33 | 73 | M | Normal | *RUNX1* | AML with MR gene mutations | Adverse |
| AML35 | 64 | F | 1q- | *KRAS, RUNX1, TET2* | AML with MR gene mutations | Adverse |
| AML73 | 79 | F | Normal | *BCOR, DNMT3A, FLT3-* TKD | AML with MR gene mutations | Adverse |
| AML145 | 45 | F | Normal | *NRAS, PTPN11, DNMT3A, ETV6, RUNX1, WT1, KMT2A*-PTD | AML with MR gene mutations | Adverse |
| AML157 | 61 | M | Normal | *FLT3*-ITD, *RUNX1, SF3B1, DNMT3A* | AML with MR gene mutations | Adverse |
| AML79 | 64 | M | Normal | *ASXL1, RUNX1, SH2B3, SRSF2, TET2* | AML with MR gene mutations | Adverse |
| AML83 | 77 | M | 12p- | *ASXL1, DNMT3A, NRAS, U2AF1, STAG2, BCOR* | AML with MR gene mutations | Adverse |
| AML85 | 63 | M | Monosomy X | *KIT, RUNX1, SF3B1, BCOR* | AML with MR gene mutations | Adverse |
| AML92 | 53 | F | Normal | *RUNX1* | AML with MR gene mutations | Adverse |
| AML94 | 74 | F | Trisomy 13 | *RUNX1* | AML with MR gene mutations | Adverse |
| AML96 | 75 | M | Normal | *SRSF2* | AML with MR gene mutations | Adverse |
| AML103 | 77 | M | Trisomy 11 | *ASXL1, EZH2, IDH2, NRAS, MLL-*PTD | AML with MR gene mutations | Adverse |
| AML14 | 48 | M | t(6;9)(p23;q34) | *FLT3*-ITD | AML with *DEK::NUP214* fusion | Adverse |
| AML71 | 57 | F | t(6;9)(p23;q34) | *WT1* | AML with *DEK::NUP214* fusion | Adverse |
| AML24 | 69 | F | Monosomy16, Monosomy 18 | *TP53, FLT3*-ITD | AML with mutated *TP53* (AML-*TP53*) | Adverse |
| AML93 | 60 | M | Normal | *TP53* | AML with mutated *TP53* (AML-*TP53*) | Adverse |
| AML32 | 78 | M | t(11;19)(q23;p13.1) | *IDH2, NRAS, WT1, KMT2A-ELL* | AML with other *KMT2A* rearrangements (*KMT2A-*r) | Adverse |
| AML62 | 36 | F | t(11;19)(q23;p13.1) | *KMT2A*-rearrangement | AML with other *KMT2A* rearrangements (*KMT2A-*r) | Adverse |
| AML102 | 19 | M | t(11;19)(q23;p13.1) | *KMT2A-*rearrangement, *WT1* | AML with other *KMT2A* rearrangements (*KMT2A-*r) | Adverse |
| AML117 | 33 | F | t(11;19)(q23;p13) | *KMT2A*-rearrangement | AML with other *KMT2A* rearrangements (*KMT2A*-r) | Adverse |
| AML141 | 21 | F | t(10;11), 1p-, 6q- | *KRAS, PHF6* | AML with other *KMT2A* rearrangements (*KMT2A*-r) | Adverse |
| AML120 | 84 | M | Monosomy 7 | *DNMT3A, RUNX1, U2AF1* | AML with MR gene mutations | Adverse |
| AML147 | 79 | M | Monosomy 7 | *MPL, RUNX1, NRAS, SRSF2, TET2, GNB1* | AML with MR gene mutations | Adverse |
| AML37 | 47 | M | 7q- | *IDH1, RUNX1* | AML with MR gene mutations | Adverse |
| AML136 | 73 | F | 5q- and 17p- | No mutations | AML with MR cytogenetic abnormalities | Adverse |
| AML154 | 84 | M | 7q- and 17p- | *JAK2, DNMT3A, TP53, EZH2, TET2, RUNX1* | AML with MR gene mutations | Adverse |
| AML27 | 25 | F | Complex (incl 17p-, +8, 9q-, 22q-) | No mutations | AML with MR cytogenetic abnormalities | Adverse |
| AML41 | 56 | M | Complex (incl-7,12p-) | *IDH2* | AML with MR cytogenetic abnormalities | Adverse |
| AML59 | 54 | M | Complex (t(1;11) (p32;q23),-1) | *U2AF1, KMT2A*-rearrangement | AML with MR gene mutations | Adverse |
| AML66 | 77 | M | Complex (t(1;9)(q32;q34), +8, -1) | *RUNX1, DNMT3A, BCR-ABL* | AML with MR gene mutations | Adverse |
| AML38 | 72 | M | Complex (incl -5, 7q-, 12p-) | *TP53* | AML with mutated *TP53* (AML-*TP53*) | Adverse |
| AML39 | 59 | M | Complex (incl 5q-, 17p-) | *TP53* | AML with mutated *TP53* (AML-*TP53*) | Adverse |
| AML49 | 21 | F | Complex (t(1;17), t(3;8), del (7)) | *TP53* | AML with mutated *TP53* (AML-*TP53*) | Adverse |
| AML53 | 58 | M | Complex (incl 5q-, -7) | *TP53* | AML with mutated *TP53* (AML-*TP53*) | Adverse |
| AML55 | 40 | F | Complex (incl 5q-, 7q-) | *TP53, TET2* | AML with mutated *TP53* (AML-*TP53*) | Adverse |
| AML161 | 76 | F | Complex (5q-, +8, 17p-) | *TP53, DNMT3A* | AML with mutated *TP53* (AML-*TP53*) | Adverse |
| AML125 | 66 | F | Complex (inv(3)(q21;q26), +8,inv(12)(p11q1)) | *GATA2, MECOM*-rearrangement, *SF3B1, ETV6, NTRK3* | AML with *GATA2::MECOM* | Adverse |
| AML76 | 52 | F | inv(3)(q21;q26) | *PTPN11, SF3B1* | AML with *GATA2::MECOM* | Adverse |
| AML109 | 66 | M | inv(3)(q21;q26) | No mutations | AML with *GATA2::MECOM* | Adverse |
| AML114 | 75 | M | inv(3)(q21;q26) | *KRAS, RUNX1, WT1* | AML with *GATA2::MECOM* | Adverse |

**Supplementary Table 2.** Comparison of AML sample classifications using the International Consensus Classification (ICC) between Arber DA *et al* (14) and our study (*in vitro* and *in vivo*) showed no statistically significant differences (*P* = 0.9; statistical analysis was done by The Kolmogorov-Smirnov test).

| ICC AML categories | Samples used in Arber DA *et al* study  (n=694) | Samples used in our study (n=100) |
| --- | --- | --- |
| AML with *RUNX1::RUNX1T1* fusion | 41 (5.9%) | 3 (3%) |
| AML with *CBFB::MYH11* fusion | 45 (6.5%) | 5 (5%) |
| AML with *DEK::NUP214* fusion | 15 (2.2%) | 2 (2%) |
| AML with *KMT2A::MLLT3* | 26 (3.7%) | 2 (2%) |
| AML with other *KMT2A* rearrangements (*KMT2A*-r) | 14 (2%) | 5 (5%) |
| AML with *GATA2::MECOM* | 36 (5.2%) | 4 (4%) |
| AML with other *MECOM* rearrangements (*MECOM*-r) | 21 (3%) | 0 |
| AML with mutated *NPM1* | 170 (24.5%) | 26 (26%) |
| AML with in-frame bZIP *CEBPA* mutations | 47 (6.8%) | 2 (2%) |
| AML with mutated *TP53* (AML-*TP53*) | 52 (7.5%) | 8 (8%) |
| AML with MR gene mutations | 174 (25.1%) | 24 (24%) |
| AML with MR cytogenetic abnormalities | 19 (2.7%) | 5 (5%) |
| AML, not otherwise specified (AML, NOS) | 34 (4.9%) | 14 (14%) |

MR: myelodysplasia-related

**Supplementary Table 3.** Different genomic profiles in men and women from AML17 and AML19 trials where complete genomic workup was available.

| **Characteristic** | **Female**  **N = 1978** | **Male**  **N = 2303** | **p-value** |
| --- | --- | --- | --- |
| **Cytogenetic risk** |  |  | 0.013 |
| Favorable | 209 (11%) | 249 (11%) |  |
| Intermediate | 1413 (71%) | 1559 (68%) |  |
| Adverse | 356 (18%) | 495 (21%) |  |
|  |  |  |  |
| ***NPM1*** |  |  | <0.001 |
| Mutant | 700 (35%) | 539 (23%) |  |
| Wild type | 1278 (65%) | 1764 (77%) |  |
|  |  |  |  |
| ***FLT3*-ITD** |  |  | <0.001 |
| Mutant | 430 (22%) | 370 (16%) |  |
| Wild Type | 1548 (78%) | 1933 (84%) |  |
|  |  |  |  |

**Supplementary Table 4.** Characteristics of acute myeloid leukemia patients used for immunohistochemistry.

| Number of patients | 140 |
| --- | --- |
| Median age in years (range) | 61 (18 – 93) |
| Sex ratio (Male: Female) | 1.3:1 |
| MRC Cytogenetic risks: |  |
| - Favorable | 14 (10%) |
| - Intermediate   - *FLT3*-ITD and *NPM1* mutated 16   - *FLT3*-ITD and *NPM1* wild-type 22   - *FLT3*-WT and *NPM1* mutated 24   - *FLT3*-WT and *NPM1* wild-type 28 | 94 (67%) |
| - Adverse | 14 (10%) |
| - No record | 18 (13%) |

**Supplementary Table 5.** Multivariate (Cox regression) analysis of patients with intermediate and high AR expressions.

|  | **OS** | | | |
| --- | --- | --- | --- | --- |
| **Variable** | | **HR** | **95% CI** | ***P* value** |
| **Age** | | 0.99 | 0.97 - 1.01 | 0.37 |
| **Sex** | | 1.26 | 0.59 - 2.67 | 0.55 |
| **AR expression** | |  |  |  |
| - Intermediate (n = 38) | | 1.62 | 0.75 - 3.47 | 0.22 |
| - High (n = 25) | | 2.66 | 1.00 - 7.02 | **0.04*** |
| **MRC cytogenetic risk group** | |  |  |  |
| - Intermediate (n = 63) | | 2.20 | 0.84 - 5.78 | 0.11 |
| - Adverse (n = 10) | | 6.27 | 1.92 - 20.51 | **0.002*** |

**Supplementary Table 6:** List of primary antibodies used in Western blot analysis.

| **Antigen** | **Host** | **Dilution** | **Supplier** | **Catalog no.** |
| --- | --- | --- | --- | --- |
| **Androgen receptor** | Rabbit | 1:2000 | Cell Signaling Technology | 5153 |
| **Estrogen receptor α** | Rabbit | 1:1000 | Cell Signaling Technology | 13258 |
| **Estrogen receptor β** | Rabbit | 1:1000 | Cell Signaling Technology | 89954 |
| **Progesterone receptor** | Rabbit | 1:1000 | Cell Signaling Technology | 8757 |
| **Alpha tubulin** | Mouse | 1:2000 | Cell Signaling Technology | 3873 |
| **Beta actin** | Rabbit | 1:2000 | Cell Signaling Technology | 4970 |

**Supplementary Table 7:** List of antibodies used in flow cytometry analysis.

| Antibody | Fluorochrome | Clone | Catalog no. | Supplier |
| --- | --- | --- | --- | --- |
| Mouse CD45 | PerCP-Cy5.5 | 30F-11 | 45-0451-82 | Thermo Fisher Scientific |
| Human CD3 | APC | HIT3a | 555342 | BD Biosciences |
| Human CD19 | FITC | HIB19 | 555412 | BD Biosciences |
| Human CD33 | APC | WM53 | 551378 | BD Biosciences |
| Human CD33 | PE | WM53 | 555450 | BD Biosciences |
| Human CD34 | PC7 | 581 | A21691 | Beckman Coulter |
| Human CD38 | APC-A700 | LS198-4-3 | B23489 | Beckman Coulter |
| Human CD45 | APC | J33 | IM2473 | Beckman Coulter |
| Human CD45 | PE | J33 | A07783 | Beckman Coulter |
| Human CD45RA | PE | ALB11 | IM1834U | Beckman Coulter |
| HLA-DR | FITC | - | 555811 | BD Biosciences |

**REFERENCES**

1. Moilanen AM, Riikonen R, Oksala R, Ravanti L, Aho E, Wohlfahrt G, et al. Discovery of ODM-201, a new-generation androgen receptor inhibitor targeting resistance mechanisms to androgen signaling-directed prostate cancer therapies. Scientific reports. 2015;5:12007.

2. Burnett AK, Milligan D, Prentice AG, Goldstone AH, McMullin MF, Hills RK, et al. A comparison of low-dose cytarabine and hydroxyurea with or without all-trans retinoic acid for acute myeloid leukemia and high-risk myelodysplastic syndrome in patients not considered fit for intensive treatment. Cancer. 2007;109(6):1114-24.

3. Taussig DC, Miraki-Moud F, Anjos-Afonso F, Pearce DJ, Allen K, Ridler C, et al. Anti-CD38 antibody-mediated clearance of human repopulating cells masks the heterogeneity of leukemia-initiating cells. Blood. 2008;112(3):568-75.

4. Miraki-Moud F, Ghazaly E, Ariza-McNaughton L, Hodby KA, Clear A, Anjos-Afonso F, et al. Arginine deprivation using pegylated arginine deiminase has activity against primary acute myeloid leukemia cells in vivo. Blood. 2015;125(26):4060-8.

5. Wang Q, Carroll JS, Brown M. Spatial and temporal recruitment of androgen receptor and its coactivators involves chromosomal looping and polymerase tracking. Molecular cell. 2005;19(5):631-42.

6. Altıntaş UB, Seo JH, Giambartolomei C, Ozturan D, Fortunato BJ, Nelson GM, et al. Decoding the epigenetics and chromatin loop dynamics of androgen receptor-mediated transcription. Nature communications. 2024;15(1):9494.

7. Andrews S. FastQC: A Quality Control Tool for High Throughput Sequence Data <http://wwwbioinformaticsbabrahamacuk/projects>. 2010.

8. Bolger AM, Lohse M, Usadel B. Trimmomatic: a flexible trimmer for Illumina sequence data. Bioinformatics. 2014;30(15):2114-20.

9. Dobin A, Davis CA, Schlesinger F, Drenkow J, Zaleski C, Jha S, et al. STAR: ultrafast universal RNA-seq aligner. Bioinformatics. 2012;29(1):15-21.

10. Putri GH, Anders S, Pyl PT, Pimanda JE, Zanini F. Analysing high-throughput sequencing data in Python with HTSeq 2.0. Bioinformatics. 2022;38(10):2943-5.

11. Love MI, Huber W, Anders S. Moderated estimation of fold change and dispersion for RNA-seq data with DESeq2. bioRxiv. 2014:002832.

12. Gentleman RC, Carey VJ, Bates DM, Bolstad B, Dettling M, Dudoit S, et al. Bioconductor: open software development for computational biology and bioinformatics. Genome Biology. 2004;5(10):R80.

13. Benjamini Y, Hochberg Y. Controlling the false discovery rate: a practical and powerful approach to multiple testing. Journal of the royal statistical society series b-methodological. 1995;57:289-300.

14. Arber DA, Orazi A, Hasserjian RP, Borowitz MJ, Calvo KR, Kvasnicka HM, et al. International Consensus Classification of Myeloid Neoplasms and Acute Leukemias: integrating morphologic, clinical, and genomic data. Blood. 2022;140(11):1200-28.
